# Identifying Regional Variation in the Prevalence of Postpartum Haemorrhage: A Systematic Review and Meta-Analysis

**DOI:** 10.1371/journal.pone.0041114

**Published:** 2012-07-23

**Authors:** Clara Calvert, Sara L. Thomas, Carine Ronsmans, Karen S. Wagner, Alma J. Adler, Veronique Filippi

**Affiliations:** London School of Hygiene and Tropical Medicine, London, United Kingdom; Lerner Research Institute, Cleveland Clinic, United States of America

## Abstract

**Objective:**

To provide regional estimates of the prevalence of maternal haemorrhage and explore the effect of methodological differences between studies on any observed regional variation.

**Methods:**

We conducted a systematic review of the prevalence of maternal haemorrhage, defined as blood loss greater than or equal to 1) 500 ml or 2) 1000 ml in the antepartum, intrapartum or postpartum period. We obtained regional estimates of the prevalence of maternal and severe maternal haemorrhage by conducting meta-analyses and used meta-regression to explore potential sources of between-study heterogeneity.

**Findings:**

No studies reported the prevalence of antepartum haemorrhage (APH) according to our definitions. The prevalence of postpartum haemorrhage (PPH) (blood loss ≥500 ml) ranged from 7.2% in Oceania to 25.7% in Africa. The prevalence of severe PPH (blood loss ≥1000 ml) was highest in Africa at 5.1% and lowest in Asia at 1.9%. There was strong evidence of between-study heterogeneity in the prevalence of PPH and severe PPH in most regions. Meta-regression analyses suggested that region and method of measurement of blood loss influenced prevalence estimates for both PPH and severe PPH. The regional patterns changed after adjusting for the other predictors of PPH indicating that, compared with European women, Asian women have a lower prevalence of PPH.

**Conclusions:**

We found evidence that Asian women have a very low prevalence of PPH compared with women in Europe. However, more reliable estimates will only be obtained with the standardisation of the measurement of PPH so that the data from different regions are comparable.

## Introduction

Haemorrhage is a leading cause of maternal death worldwide, accounting for over 30% of maternal deaths in Africa and Asia [1]. Furthermore, it is a substantial source of maternal morbidity and can have long-term effects on a woman’s health [2]. Untreated maternal haemorrhage is associated with adverse health consequences such as renal failure and anaemia and may detrimentally affect a woman’s psychological well-being [3], [4]. In very severe cases hysterectomy may be used to control the bleeding.

Maternal haemorrhage can occur in the antepartum, intrapartum or postpartum period. The WHO defines postpartum haemorrhage (PPH) as blood loss of 500 ml or more from the genital tract after delivery, although some studies define PPH as blood loss greater than or equal to 1000 ml as this has greater clinical significance [2]. Similar definitions do not exist for antepartum haemorrhage (APH) or intrapartum haemorrhage (IPH).

Several studies have attempted to estimate the global burden of PPH. AbouZahr estimated severe PPH (blood loss ≥1000 ml) to have a global prevalence of 10.5% amongst women who had a live birth in the year 2000 [2]. In a systematic review of studies published between 1997 and 2002, supplemented by a less comprehensive search of the literature between 2003 and 2006, Carroli *et al.* found a global prevalence of PPH ≥500 ml of 6.09% and of PPH ≥1000 ml of 1.86%, much lower than AbouZahr’s estimate [5]. This review also examined the prevalence of PPH by region revealing marked differences. The prevalence of PPH ≥500 ml ranged from 2.55% in Asia to 10.45% in Africa. However, Carroli *et al.* found high between-study heterogeneity within each region.

It is important to understand whether regional variation in the prevalence of haemorrhage is likely to be due to true variation and/or due to methodological differences between studies. Factors that have been found to influence the measured prevalence of maternal haemorrhage include the method of blood loss measurement [6], [7], management of the third stage of labour [8] and whether the study is population-based or facility-based [5].

The aim of this review is to update the estimates of the global burden of PPH, expand these estimates to include APH and IPH and to examine whether similar patterns of regional variation found by Carroli *et al.* are observed. Unlike previous studies, we also examine potential sources of between-study heterogeneity using meta-regression techniques.

## Methods

This study was part of a wider systematic review of the burden of maternal haemorrhage and the main causes of maternal haemorrhage. A brief review protocol was developed and reviewed by external experts. The primary outcome of interest for this study was the prevalence of maternal haemorrhage, which includes PPH, IPH and APH. Two definitions were used for maternal haemorrhage: blood loss ≥500 ml or blood loss ≥1000 ml.

### Search Strategy

Potentially relevant articles for the systematic review were identified by searching bibliographical databases (Medline, EMBASE, Popline, and CAB abstracts) and the WHO regional databases (African Index Medicus, Eastern Mediterranean Region Index Medicus, Western Pacific Region Index Medicus and Latin American and Caribbean Center on Health Sciences Information). A full search strategy for each database was developed using MeSH and free-text words for haemorrhage and its causes and a more targeted search strategy comprised terms for APH and IPH (Appendix 1). The search strategy aimed at complementing the systematic review conducted by Carroli *et al.* between 1997 and 2002. Because Carroli *et al*. only searched for PPH studies, we screened all studies they included in the present review, and added search terms for APH and IPH for the period 1997–2002. The full search strategy was applied to articles published between 2003 and 2009, for which no previous review was available. There were no language restrictions. Additional publications were identified through manual searching of reference lists from relevant articles.

### Inclusion and Exclusion Criteria

Two authors (KW and CC) sequentially screened titles and abstracts of identified citations for potential inclusion in the review and full texts were sought for articles deemed to be relevant. Studies were eligible for inclusion if they reported dates for data collection, included data from 1990 onwards and had a sample size of more than 30 pregnant women. Trials, cohort studies, cross-sectional studies and population based case-control studies were all eligible for inclusion. To ensure that studies were representative of the population, hospital-based studies were only included if the region in which the study was conducted had ≥95% of births attended by a skilled birth attendant as identified using Demographic and Health Surveys and data compiled by the WHO [9]. Studies that relied on maternal self-reports of blood loss were not included, as such reports do not provide valid estimates of the prevalence of haemorrhage [10], [11].

Analyses were conducted on studies which reported the prevalence of maternal haemorrhage. Studies which only included caesarean section births were excluded as in many settings only a minority of births are delivered by caesarean sections, and such studies could inflate estimates of PPH as caesarean sections generally lead to higher blood loss.

### Data Extraction

Data were extracted for each paper by a single author (KW, CC or VF) on: location of study, study dates, study design, the study population, mode and management of delivery, the case definition of maternal haemorrhage, method of measurement of maternal haemorrhage and the prevalence of maternal haemorrhage. Information was also extracted on whether the study population included all women or only women at low risk of PPH. The study population was classified as at low risk of PPH if women with risk factors for PPH, such as placenta praevia or PPH in a previous delivery, were excluded. If more than one article provided data on the same population and study period, data were extracted from the article with the longest study duration. The intervention and control arms of trials were extracted separately and treated as separate datasets in the analysis.

### Data Analysis

Analyses were carried out using R 2.12.2 and Stata 11.0. The variance of each dataset’s prevalence was used to weight estimates from each study to produce pooled estimates. To prevent the prevalence of haemorrhage influencing the weight allocated to each dataset, the prevalence from each dataset was transformed using a Freeman-Tukey type arcsine square-root transformation [12], [13] and the variance was calculated as 1/(n+1). The I^2^ statistic was calculated as a measure of the proportion of the overall between-study variation in the prevalence of haemorrhage that was due to differences between the studies and not chance [14]. The DerSimonian-Laird random effects method [15] was used to combine study estimates. Estimates were stratified using regional divides consistent with those used by Carroli *et al.* [5]: Africa, Asia (including the Middle East), Europe, Northern America, Oceania and Latin America and the Caribbean. Estimates were also stratified by parity and by singleton or multiple births.

A meta-regression was conducted to identify sources of between-study heterogeneity using Stata 11.0. The prevalence of PPH for each study was transformed into an odds before conducting the meta-regression. Five potential sources of heterogeneity, which were specified *a priori,* were examined: region, mode of delivery (vaginal/vaginal and caesarean deliveries), management of the third stage of labour (active/mixed/unclear/expectant/unknown), blood loss measurement method (objectively/subjectively/both/unknown) and the study location (home and primary medical centre/hospital/population). Studies were classified as using objective measurement of blood loss if they used a calibrated collection drape, a measuring jug or weighed blood soaked swabs and linen. Studies were only considered as using active management if they described using 1) a prophylactic uterotonic, including misoprostol, 2) early cord clamping and cutting and 3) controlled cord traction to deliver the placenta [16]. The unclear category was for studies that described using one or two components of active management but did not state whether the other components of active management were used. To be classified as population-based, the study had to include all women giving birth, or a random sample of these women, from a well defined geographical catchment population, for example a city or a region. A multivariable meta-regression model was built by entering region into the model initially. As any observed association between region and prevalence of haemorrhage may be explained by the other potential sources of heterogeneity, these were added sequentially into the model starting with the variable which showed the strongest association with prevalence of PPH on univariable analysis; a variable remained in the multivariable model if it was independently associated with the prevalence of maternal haemorrhage at p≤0.10.

Sensitivity analyses were conducted by restricting to only studies which used objective methods for measuring blood loss.

## Results

**Figure 1:**
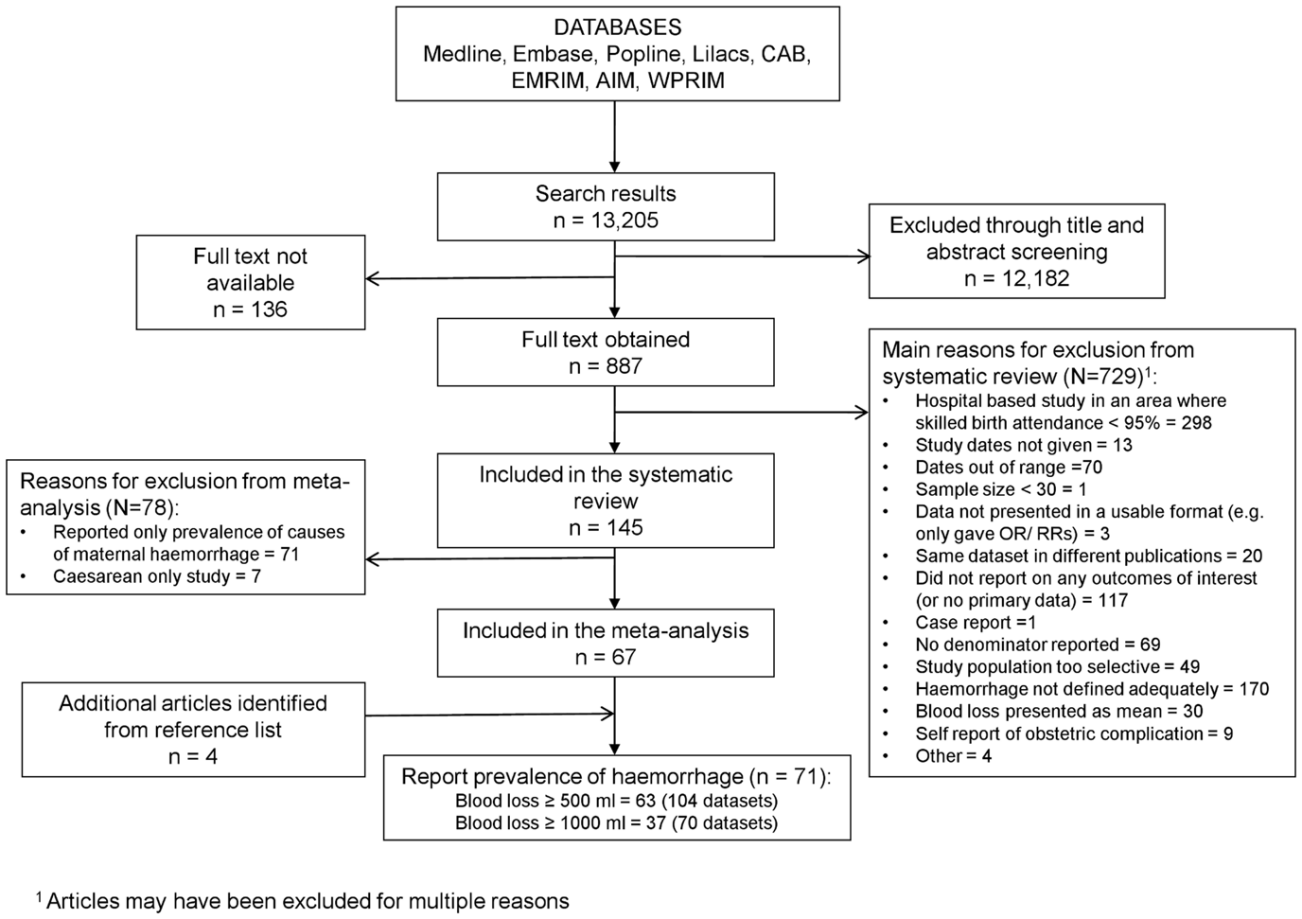
Study selection for inclusion in the systematic review and meta-analysis.

We identified 13,205 potentially relevant articles and included 145 in the systematic review, of which 67 reported the prevalence of maternal haemorrhage (Figure 1). An additional four eligible articles were identified through manual searches of reference lists. A total of 71 articles, providing 123 datasets were analysed (references provided in File S1). No studies reported the prevalence of APH as blood loss ≥500 ml or ≥1000 ml. We classified all included studies as measuring PPH, although there was variation in the timings of blood loss measurement. Whilst many of the studies were restricted to blood loss after delivery, three studies measured blood loss both during and after delivery [17]–[19]; two of these studies stated that they were measuring PPH [18], [19]. Thirty-one studies stated that they measured PPH but did not specify the timing of blood loss measurement (for example: [20], [21]). A further four studies referred to the outcome as “blood loss” without stating specifically that they were measuring PPH or specifying the timings of blood loss measurement [22]–[25], and two studies measured blood loss at delivery [26], [27].

Within the 123 datasets, 104 datasets defined PPH as blood loss ≥500 ml; 39 were from Asia, 27 from Europe, 13 from Oceania, ten from Northern America, nine from Latin America and the Caribbean and six from Africa. Seventy datasets defined PPH as blood loss ≥1000 ml; 28 were from Europe, 23 from Asia, seven from Latin America and the Caribbean, six from Northern America, four from Africa and two from Oceania. Fifty-one datasets reported data for both definitions. Table 1 summarises the characteristics of the datasets for each region. The proportion of datasets which had a trial study design varied from 46.3% in Oceania to 100% in Africa and the proportion with a sample size >1000 women ranged from 0 in Africa to 55.5% in Latin America and the Caribbean. Women were classified as at low risk of PPH in 27.3% of datasets in Latin America and the Caribbean with increasing proportions up to 47.4% in Europe.

**Table 1:** Description of datasets in each region.

		Africa	Latin America and Caribbean	Northern America	Asia	Europe	Oceania
**Total number of datasets**		6	11	12	43	38	13
**Study characteristics**							
Study design	Observational	0 (0)	2 (18.2)	4 (33.3)	10 (23.3)	17 (44.7)	7 (53.8)
	Trial	6 (100)	9 (81.8)	8 (66.7)	33 (76.7)	21 (55.3)	6 (46.2)
Sample Size	≤1000	6 (100)	5 (45.5)	9 (75.0)	34 (79.1)	20 (52.6)	6 (46.2)
	>1000	0 (0)	6 (55.5)	3 (25.0)	9 (20.9)	18 (47.4)	7 (53.8)
Method of measurement of blood loss	Objectively	4 (66.7)	6 (54.6)	0 (0)	29 (67.4)	12 (31.6)	0 (0)
	Subjectively	2 (33.3)	4 (36.4)	12 (100.0)	7 (16.3)	9 (23.7)	5 (38.5)
	Both	0 (0)	0 (0)	0 (0)	2 (4.7)	5 (13.2)	2 (15.4)
	Unknown	0 (0)	1 (9.1)	0 (0)	5 (11.6)	12 (31.6)	6 (46.2)
Study location	Population	0 (0)	2 (18.2)	1 (8.3)	1 (2.3)	6 (15.8)	5 (38.5)
	Hospital	0 (0)	9 (81.8)	9 (75.0)	40 (93.0)	29 (76.3)	6 (46.2)
	Primary medical centre/home	6 100.0)	0 (0)	2 (16.7)	2 (4.7)	3 (7.9)	2 (15.4)
**Characteristics of Women**							
Study population	All women	4 (66.7)	8 (72.7)	5 (41.7)	24 (55.8)	20 (52.6)	9 (69.2)
	Low PPH risk	2 (33.3)	3 (27.3)	7 (58.3)	19 (44.2)	18 (47.4)	4 (30.8)
Include multiple gestation	Yes	2 (33.3)	1 (9.1)	2 (16.7)	0 (0)	3 (7.9)	4 (30.7)
	No	0 (0)	8 (72.7)	7 (58.3)	25 (58.1)	18 (47.4)	5 (38.5)
	Not specified	4 (66.7)	2 (18.2)	3 (25.0)	18 (41.9)	17 (44.7)	4 (30.8)
**Management of delivery**							
	Active	0 (0)	4 (36.4)	6 (50.0)	23 (53.5)	17 (34.0)	0 (0)
	Mixed	4 (66.7)	2 (18.2)	0 (0)	2 (4.7)	2 (20.0)	0 (0)
	Unclear^1^	0 (0)	1 (9.1)	3 (25.0)	9 (20.9)	2 (20.0)	2 (15.4)
	Expectant	2 (33.3)	0 (0)	0 (0)	1 (2.3)	3 (7.9)	0 (0)
	Unknown	0 (0)	4 (36.4)	3 (25.0)	8 (18.6)	14 (36.8)	11 (84.6)

### Prevalence of Postpartum Haemorrhage

The pooled prevalence of PPH ≥500 ml overall and stratified by region, parity and singleton/multiple births is presented in table 2, with individual prevalence estimates from the contributing studies outlined in supplementary Figures S1, S2, S3, S4, S5, S6. Overall, 10.8% of women were estimated to suffer PPH (95% CI: 9.6–12.1). However, there was wide regional variation in PPH prevalence, ranging from 7.2% of women giving birth in Oceania (95% CI: 6.3–8.1) to 25.7% in Africa (95% CI: 13.9–39.7). Just over 8% of women giving birth were estimated to suffer from PPH ≥500 ml in both Latin America and Asia and prevalence was approximately 13% in Europe and in Northern America. Within all regions there was strong evidence for between-study heterogeneity (I^2^>95%, p<0.001). Further breakdown of the regional estimates of PPH ≥500 ml are provided in Supplementary Table S1.

**Table 2:** Prevalence of PPH ≥500 ml.

	No. articles	No. datasets	No. womengiving birth	No. women with blood loss ≥500 ml	I^2^	P-Value^1^	Prevalence of blood loss ≥500 ml per 100 womengiving birth	Prevalence of blood loss ≥500 ml per 100 women giving birth based only on studies which used objective method of blood loss measurement
**Overall**	63	104	1,003,694	89,378	99.7	<0.001	10.8 (9.6–12.1)	14.2 (11.5–17.1)
**Region**								
**Africa**	3	6	2,738	645	98.4	<0.001	25.7 (13.9–39.7)	28.0 (20.7–24.5)
**Latin America and the Caribbean**	6	9	23,129	2,076	96.2	<0.001	8.2 (6.1–10.4)	11.9 (6.0–19.5)
**Northern America**	7	10	28,580	5,934	99.5	<0.001	13.1 (6.3–21.7)	NA
**Asia**	20	39	215,611	9,494	97.5	<0.001	8.5 (7.1–10.1)	10.0 (8.2–12.0)
**Europe**	18	27	378,617	51,204	99.5	<0.001	12.7 (10.1–15.6)	22.9 (15.2–31.6)
**Oceania**	9	13	355,019	20,025	98.3	<0.001	7.2 (6.3–8.1)	NA
**Number of foetuses** 2								
**Singleton only**	36	62	275053	22078	99.5	<0.001	10.6 (8.6–12.7)	–
**Multiple only**	2	2	67	20	99.7	<0.001	32.4 (11.7–57.7)	–
**Both**	5	10	170234	11124	92.7	<0.001	7.2 (6.4–8.1)	–
**Not specified**	22	32	558340	56156	99.8	<0.001	12.74 (9.9–15.8)	–
**Parity** 2,3								
**Nulliparous**	11	14	225444	29562	99.8	<0.001	12.9 (9.1–17.4)	–
**Multiparous**	8	9	302951	29045	99.9	<0.001	10.0 (6.4–14.3)	–
**Both**	38	73	260562	21385	99.2	<0.001	10.7 (9.2–12.3)	–
**Not specified**	12	15	124318	9337	98.9	<0.001	10.1 (7.7–12.7)	–

1 From test of heterogeneity.

2 Where studies presented PPH prevalence by parity or gestation the data set was disaggregated into two data sets and consequently the total number of data sets is higher for parity and gestation that the total number for the overall estimate.

3 In one of the studies which stratified PPH prevalence by parity there was some information missing for parity for some of the women and so they were excluded from this analysis; consequently the total number of women giving birth for the parity analysis is slightly lower than the women used in the overall analysis.

Women who had multiple births had a higher prevalence of PPH ≥500 ml compared with women who had singleton births, at 32.4% (95% CI: 11.7–57.7) vs. 10.6% (95% CI: 8.6–12.7) respectively (Table 1). Women who were having their first baby had a prevalence of PPH of 12.9% (95% CI: 9.1–17.4) compared with 10.0% amongst women who were multiparous (95% CI: 6.4–14.3).

### Prevalence of Severe Postpartum Haemorrhage

Whilst the overall prevalence of severe PPH ≥1000 ml, was much lower (2.8%, 95% CI: 2.4–3.2) than the prevalence of PPH, similar regional patterns were observed (Table 3 and Supplementary Figures S7, S8, S9, S10, S11, S12). Africa had the highest prevalence of severe PPH at 5.1% (95% CI: 0.3–15.3), followed by a prevalence of 4.3% in Northern America, with the lowest prevalence in Asia at 1.9%. Around 3% of women giving birth in Latin America, Europe and Oceania were estimated to suffer severe PPH. There was strong evidence for between-study heterogeneity within most regions (p<0.001), but not for the six Northern American studies (I^2^ = 2.2%, p = 0.4) and the two Oceania studies (I^2^ = 0%, p<0.68). Sub-regional estimates of PPH ≥1000 ml are presented in Supplementary Table S2.

**Table 3:** Prevalence of PPH ≥1000 ml.

	No. articles	No. datasets	No. women giving birth	No. women with blood loss ≥1000 ml	I^2^	P-Value^1^	Prevalence of blood loss ≥1000 ml per 100 women giving birth	Prevalence of blood loss ≥1000 ml per 100 women giving birth based only on studies which used objective method of blood loss measurement
**Overall**	37	70	503,046	6,505	97.8	<0.001	2.8 (2.4–3.2)	4.2 (3.2–5.3)
**Region**								
**Africa**	2	4	1,889	99	98.2	<0.001	5.1 (0.3–15.3)	5.1 (0.3–15.3)
**Latin America and the Caribbean**	4	7	15,551	386	93.9	<0.001	3.3 (1.8–5.2)	4.1 (1.9–7.0)
**Northern America**	4	6	21,744	939	2.2	0.40	4.3 (3.9–4.68)	NA
**Asia**	11	23	11,416	293	88.6	<0.001	1.9 (1.2–2.8)	2.5 (1.7–3.4)
**Europe**	18	28	452,116	4,779	98.0	<0.001	2.8 (2.3–3.4)	6.8 (5.2–8.4)
**Oceania**	1	2	330	9	0	0.68	3.0 (1.4–5.1)	NA
**Number of foetuses** 2								
**Singleton only**	19	32	139584	2120	98.5	<0.001	2.8 (1.9–3.6)	–
**Multiple only**	2	2	67	3	0	0.97	5.7 (1.5–12.4)	–
**Both**	2	4	2228	25	72.1	0.01	1.1 (0–2.1)	–
**Not specified**	16	34	361167	4357	96.6	<0.001	3.0 (2.5–3.6)	–
**Parity** 2								–
**Nulliparous**	3	3	8151	311	97.7	<0.001	5.3 (2.3–9.4)	–
**Multiparous**	2	2	7211	193	99.4	<0.001	9.6 (0–39.3)	–
**Both**	25	55	325296	5024	96.9	<0.001	3.0 (2.3–3.7)	–
**Not specified**	9	12	161317	977	92.8	<0.001	1.3 (0.1–1.6)	–

1 From test of heterogeneity.

2 Where studies presented PPH prevalence by parity or gestation the data set was disaggregated into two data sets and consequently the total number of data sets is higher for parity and gestation that the total number for the overall estimate.

When severe PPH ≥1000 ml was stratified singleton/multiple deliveries very similar patterns to PPH ≥500 ml were observed (Table 2). Severe PPH was higher for multiple births (5.7%, 95% CI: 1.5–12.4) compared with singleton births (2.8%, 95% CI: 1.9–3.6). However a different pattern was observed for parity, whereby multiparous women has a higher prevalence of severe PPH (9.6%, 95% CI: 0–39.3) compared with women giving birth for the first time (5.3%, 95% CI: 2.3–3.7).

### Sources of Heterogeneity

In the univariate meta-regression there was evidence that region (p = 0.01), blood loss measurement method (p<0.001), type of management of deliveries (p = 0.002), mode of delivery (p = 0.05) and study location (p = 0.03) influenced the between-study variation in the prevalence of PPH of blood loss ≥500 ml (Table 4). In the multivariable meta-regression model, region (p = 0.03), method of measurement of blood loss (p<0.001) and, to some extent, management of delivery (p = 0.10) remained independently associated with prevalence. Together these three predictors explained 30% of the between-study heterogeneity. Studies where expectant management of deliveries was used had, on average, a higher prevalence of PPH compared with studies that used active management of labour. Studies that used subjective measurement of blood loss and those not stating how blood loss was measured had, on average, a lower prevalence of PPH than studies using objective measurements. Introducing measurement of blood loss and management of delivery into the model with region reduced the strength of the association between region and prevalence of PPH. In this multivariable model, we found that compared with Europe, studies conducted in Asia have, on average, a lower prevalence of PPH. Mode of delivery was not included in the final model as it was strongly correlated with management of delivery (correlation coefficient = 0.60, p<0.001) and showed less strong evidence for an association with PPH than management of delivery. In addition, eight of the nine “mixed” delivery studies did not specify how delivery was managed, and consequently the “unknown” management of delivery category captures the effect of studies which included both vaginal and caesarean deliveries.

**Table 4:** Meta-regression for PPH ≥500 ml.

		No. of datasets (n = 104)	Prevalence ofhaemorrhage (%)	Univariable		Multivariable model (30.0% of variation explained)	
				OR (95% CI)	P-value	AOR^1,2^ (95% CI)	P-value
**Region**	Europe	27	12.7 (10.1–15.6)	1		1	
	Africa	6	25.7 (13.9–39.7)	2.55 (1.15–5.64)		1.39 (0.61–3.17)	
	Latin America	9	8.2 (6.1–10.4)	0.71 (0.36–1.40)		0.67 (0.36–1.25)	
	N. America	10	13.1 (6.3–21.7)	1.05 (0.55–2.01)		1.53 (0.80–2.92)	
	Asia	39	8.5 (7.1–10.1)	0.67 (0.43–1.04)		0.61 (0.40–0.95)	
	Oceania	13	7.2 (6.3–8.1)	0.63 (0.35–1.15)	0.01	0.74 (0.42–1.30)	0.03
**Mode of delivery** 3	Mixed	27	7.8 (5.9–10.0)	1		–	
	Vaginal	77	12.1 (10.4–13.9)	1.51 (1.01–2.27)	0.05	–	
**Management of deliveries** 3	Active	34	9.5 (7.8–11.3)	1		1	
	Mixed	10	19.9 (11.8–29.6)	2.34 (1.26–4.34)		1.52 (0.77–3.01)	
	Unclear	17	10.5 (7.3–14.10)	0.93 (0.55–1.56)		0.71 (0.41–1.24)	
	Expectant	6	21.9 (15.2–29.6)	2.82 (1.31–6.04)		2.20 (1.04–4.66)	
	Unknown	37	8.6 (6.9–10.5)	0.85 (0.57–1.24)	0.002	1.31 (0.81–2.11)	0.10
**Method of measurement of blood loss**	Objectively	41	14.2 (11.5–17.1)	1		1	
	Subjectively	36	9.5 (6.7–12.6)	0.64 (0.44–0.95)		0.53 (0.34–0.82)	
	Both	6	17.5 (11.4–24.5)	1.43 (0.68–3.03)		2.25 (0.93–5.40)	
	Unknown	21	6.3 (4.5–8.4)	0.41 (0.26–0.66)	<0.001	0.35 (0.20–0.60)	<0.001
**Study location**	Population	14	8.7 (6.1–11.7)	1		–	
	Hospital	77	10.1 (8.4–12.0)	1.09 (0.65–1.84)		–	
	Primary medicalcentre/home	13	18.4 (11.2–26.8)	2.23 (1.12–4.45)	0.03	–	

1 AOR = Adjusted odds ratio.

2 Adjusted for region, management of deliveries and method of measurement of blood loss.

3 Mode of delivery and management of deliveries are correlated (correlation coefficient = 0.60, P<0.001).

There was evidence, in univariate analyses, that variation in the prevalence of severe PPH between studies was associated with mode of delivery (p = 0.08) and blood loss measurement method (p = 0.001), but not with region (p = 0.47) (Table 5). However, in a multivariable model, region (p = 0.02) and blood loss measurement method (p<0.001) were independently associated with variation in prevalence of severe PPH, together explaining 29.4% of the between-study heterogeneity. After accounting for measurement of blood loss, studies conducted in Asia had, on average, a lower prevalence of severe PPH compared to studies conducted in Europe and studies conducted in Northern America had, on average, a higher prevalence.

**Table 5:** Meta-regression for PPH ≥1000 ml.

		No. of datasets^1^(n = 69)	Prevalence ofhaemorrhage (%)	Univariable		Multivariable model (29.4% of variation explained)	
				OR (95% CI)	P-value	AOR^2,3^ (95% CI)	P-value
**Region**	Europe	27	2.8 (2.3–3.4)	1		1	
	Africa	4	5.1 (0.3–15.3)	1.16 (0.36–3.70)		0.59 (0.22–1.64)	
	Latin America	7	3.3 (1.8–5.2)	1.29 (0.51–3.22)		0.96 (0.43–2.13)	
	N. America	6	4.3 (3.9–4.7)	1.40 (0.53–3.73)		2.70 (1.06–6.85)	
	Asia	23	1.9 (1.2–2.8)	0.64 (0.35–1.18)		0.52 (0.30–0.88)	
	Oceania	2	3.0 (1.4–5.1)	1.20 (0.25–5.88)	0.47	0.97 (0.22–4.33)	0.02
**Mode of delivery**	Mixed	8	1.4 (0.6–2.5)	1		–	–
	Vaginal	61	3.1 (2.5–3.9)	2.05 (0.92–4.55)	0.08	–	–
**Management of deliveries**	Active	33	2.8 (2.1–3.6)	1		–	–
	Mixed	8	4.8 (1.8–9.3)	1.51 (0.65–3.50)		–	–
	Unclear	15	1.9 (0.8–3.2)	0.70 (0.36–1.36)		–	–
	Expectant	3	5.6 (2.1–10.6)	2.27 (0.62–8.21)		–	–
	Unknown	10	2.5 (1.4–3.9)	0.73 (0.34–1.57)	0.26	–	–
**Method of measurement** **of blood loss**	Objectively	35	4.2 (3.2–5.3)	1		1	
	Subjectively	20	1.7 (1.0–2.6)	0.41 (0.24–0.70)		0.27 (0.15–0.48)	
	Both	8	2.7 (1.4–4.4)	0.74 (0.34–1.59)		0.63 (0.28–1.44)	
	Unknown	6	0.7 (0.5–0.9)	0.24 (0.10–0.57)	0.001	0.19 (0.08–0.44)	<0.001
**Study location**	Population	3	2.1 (0.2–5.7)	1		–	–
	Hospital	8	2.7 (2.2–3.3)	1.29 (0.35–4.68)		–	–
	Primary medicalcentre/home	58	4.1 (1.1–9.1)	1.53 (0.35–6.71)	0.84	–	–

1 One dataset excluded from meta-regression as it was unclear whether it only included vaginal deliveries or whether caesarean deliveries were also included.

2 AOR = Adjusted odds ratio.

3 Adjusted for region and method of measurement of blood loss.

The prevalence of PPH was higher when the meta-analysis was restricted only to studies which used objective methods to measure blood loss. For blood loss ≥500 ml the overall prevalence was 14.2% and for blood loss ≥1000 ml the prevalence was 4.2%. A similar pattern was observed across the regions, as shown in Table 2 and Table 3.

## Discussion

Our systematic review estimates the global prevalence of PPH to be 10.8% and of severe PPH to be 2.8%. These results are higher than those estimated by Carroli *et al* [5], although, for severe PPH, lower than AbouZahr’s estimate of 10.5% [2]. It is plausible that our increased estimates of PPH prevalence compared to those of Carroli *et al.* [5] is due to a genuine increase. A study by Knight *et al.* found evidence for an increasing trend in PPH in Australia, Canada, the UK and the USA between 1991 and 2006 [28]. The authors suggest this increase could be due to rising obesity levels, changes in the management of delivery and increasing tolerance to longer duration of labour [28]. We also found good evidence for regional variation in the prevalence of PPH, although the only clear trend which emerged after other predictors of blood loss were controlled for was a particularly low prevalence in Asia. There were no clear regional trends for severe PPH at the crude level, but the pattern changed when adjusting for different blood loss measurement methods. Asian women had a very low prevalence of severe PPH compared to European women, whilst North American women had the highest prevalence.

It is possible that the lower prevalence of PPH observed amongst the Asian studies is due to regional differences in genetics or underlying risk factors. Obesity has been implicated as a risk factor for PPH [29], [30], and a substantially lower proportion of women living in Asia are obese compared with European women [31] possibly explaining some of the observed trend. Similarly, the high prevalence of severe PPH observed in America may be due to rising levels of obesity and higher maternal age in this region [29], [30].

Meta-regression techniques enabled us to examine the influence of both methodological differences between studies and of the clinical management of the deliveries on the prevalence of haemorrhage. We found that blood loss measurement method plays a critical role in the variation of reported prevalence of PPH and severe PPH, with subjective measurement leading to lower estimates of prevalence. Management of delivery was also associated with prevalence of PPH, in line with trials which have found that active management is protective against PPH [8]. However, such an association was not seen with the prevalence of severe PPH, suggesting that certain causes of severe PPH may not be amenable to prevention through active management of delivery. Taking these predictors into account affected the association between region and prevalence of both PPH and severe PPH. Amongst studies reporting PPH, blood loss measurement method and management of deliveries appeared to explain some, but not all, of the regional variation. For those studies reporting severe PPH, differences in blood loss measurement method was masking some of the regional variation, and when controlled for the association between prevalence of PPH and region became stronger. Only about 30% of the between-study heterogeneity was explained by both the final models suggesting that there are other methodological and/or biological factors explaining the remaining between-study heterogeneity. In order to reduce the chance of identifying spurious associations, study characteristics to be used in the meta-regression were specified before conducting any analyses. However, the meta-regression results still need to be interpreted with caution. The number of studies in some categories, particularly for Africa and for severe PPH, was small and this may have limited the power to detect associations. Furthermore, studies frequently did not provide adequate information on the explored sources of heterogeneity and were classified as unknown for certain categories. As with any study using observation data, these meta-regression analyses are subject to residual confounding: any associations seen between a study characteristic and the prevalence of PPH may actually reflect a true association of another unmeasured study characteristic which is correlated which the characteristic being investigated. Finally, the results of the meta-regression rely on the use of study-level covariates which may or may not reflect the relationship that would be observed if we had investigated the association at the individual level.

We did not succeed in identifying any studies which defined APH according to volume of blood loss. This is likely to be due to the fact no universal definition exists for APH. APH results from abortions, ectopic pregnancies, placenta praevia and abruptio placentae; these causes can lead to a small amount of external bleeding before labour or abortion. Placenta praevia and abruptio placentae may also cause substantial bleeding during labour. Although the WHO has defined PPH, many of the studies included did not state when blood loss was measured and others included intrapartum blood loss in the measurement of PPH.

Our review has other limitations. Firstly, for some regions there were only a few studies that may not be representative of the prevalence of PPH in the whole region. For example, none of the three studies conducted in Africa came from the northern, southern or middle regions of Africa. The African study conducted by Hoj *et al.* [32] found an extremely high prevalence of PPH which is unlikely to be representative of the whole continent. Excluding this study would have lead to a much lower estimate of the prevalence of haemorrhage in Africa. Similarly, in Asia the studies generally come from more developed regions of East Asia, such as Japan and Hong Kong, and the Middle East and these studies are unlikely to be representative of the prevalence in less economically developed counties. Secondly, there were few population based studies, with most of the studies conducted in single hospitals. Finally, there was very high between-study heterogeneity within each region and each summary regional estimate obtained from the meta-analysis is an average of within-region prevalences which are genuinely different from one another.

This work highlights the complexities in producing regional estimates of the prevalence of PPH, and calls into question whether we should be combining studies which are so methodologically varied to give one summary estimate. However, obtaining better estimates remains an important endeavour. Accurate measurements of the frequency of events such as PPH, an important cause of maternal mortality, enable assessment of progress in improving clinical practice and maternal and neonatal health outcomes. Obtaining these measurements will require increased awareness from clinicians and public health practitioners about the importance of maintaining clinical records with accurate and replicable information, and standardisation of methodologies such as the method and timing of blood loss measurement for PPH. In particular, we strongly recommend that researchers use objective methods for measuring blood loss, as opposed to visual estimation, as results from our sensitivity analysis suggest that use of non-objective methods may underestimate blood loss due to imprecise measurement. The scarcity of population representative data from developing countries highlights the need for better methods of capturing a random sample of the whole population in such areas, ultimately by capturing both births occurring within homes and facilities. Only when such data become available will we be able to provide accurate estimates of the global burden of maternal haemorrhage and ascertain whether there is true regional variation in PPH due to differences in clinical practice rather than apparent differences due to heterogeneity in the quality of studies conducted in each region.

## Supporting Information

Figure S1 **Forest plot of prevalence of PPH≥500 ml amongst studies conducted in Africa.**
(TIFF)

Figure S2 **Forest plot of prevalence of PPH≥500 ml amongst studies conducted in Latin America and the Caribbean.**
(TIFF)

Figure S3 **Forest plot of prevalence of PPH≥500 ml amongst studies conducted in Northern America.**
(TIFF)

Figure S4 **Forest plot of prevalence of PPH≥500 ml amongst studies conducted in Asia.**
(TIFF)

Figure S5 **Forest plot of prevalence of PPH≥500 ml amongst studies conducted in Europe.**
(TIFF)

Figure S6 **Forest plot of prevalence of PPH≥500 ml amongst studies conducted in Oceania.**
(TIFF)

Figure S7 **Forest plot of prevalence of PPH≥1000 ml amongst studies conducted in Africa.**
(TIFF)

Figure S8 **Forest plot of prevalence of PPH≥1000 ml amongst studies conducted in Latin America and the Caribbean.**
(TIFF)

Figure S9 **Forest plot of prevalence of PPH≥1000 ml amongst studies conducted in Northern America.**
(TIFF)

Figure S10 **Forest plot of prevalence of PPH≥1000 ml amongst studies conducted in Asia.**
(TIFF)

Figure S11 **Forest plot of prevalence of PPH≥1000 ml amongst studies conducted in Europe.**
(TIFF)

Figure S12 **Forest plot of prevalence of PPH≥1000 ml amongst studies conducted in Oceania.**
(TIFF)

Table S1 **Prevalence of PPH≥500 ml by UN regions.**
(DOCX)

Table S2 **Prevalence of PPH≥1000 ml by UN regions.**
(DOCX)

File S1 (DOCX)
