# Supplementary figures and images for: Identifying Regional Variation in the Prevalence of Postpartum Haemorrhage: A Systematic Review and Meta-Analysis
Source: PLoS One. 2012 Jul 23;7(7):e41114. doi: 10.1371/journal.pone.0041114 (PMC3402540; doi:10.1371/journal.pone.0041114)

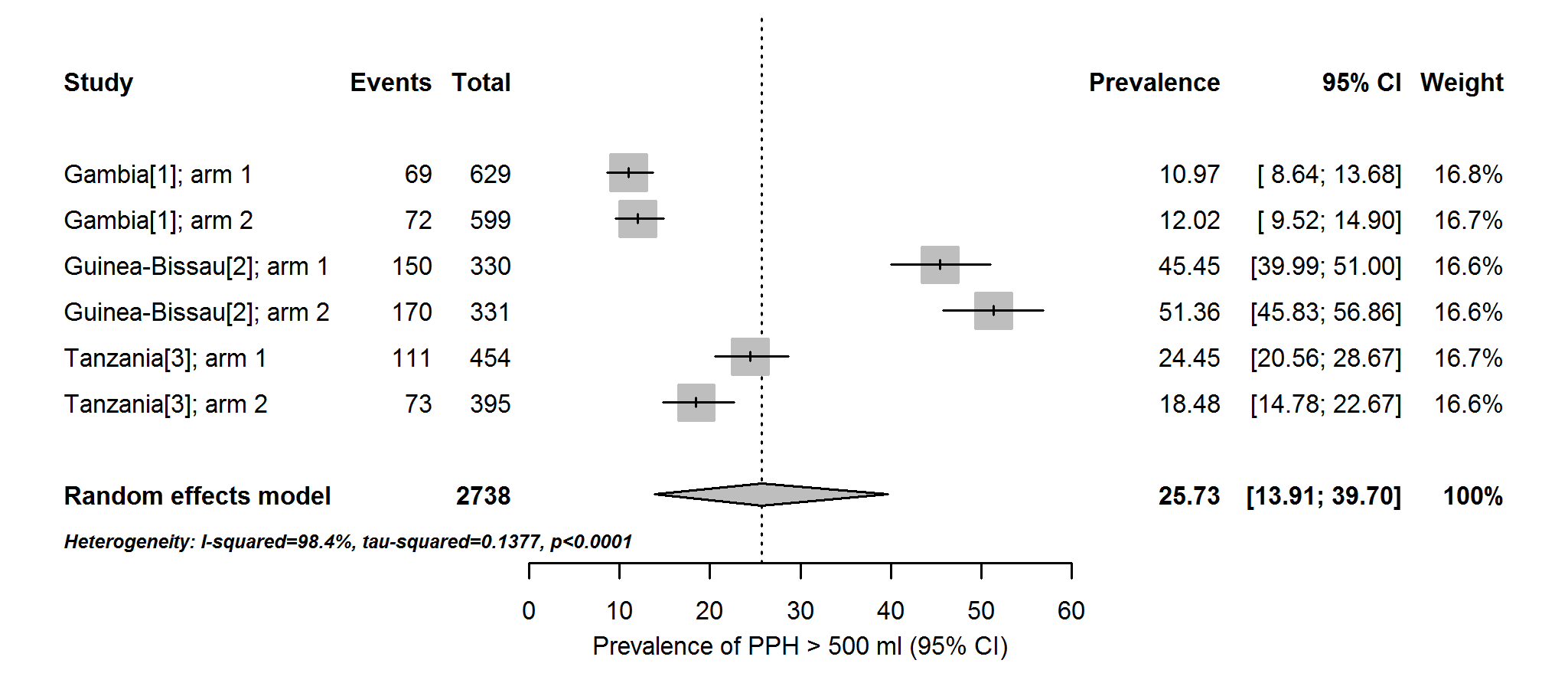

Supplement: Figure S1 — Forest plot of prevalence of PPH≥500 ml amongst studies conducted in Africa. (TIFF) [file pone.0041114.s001.tiff]

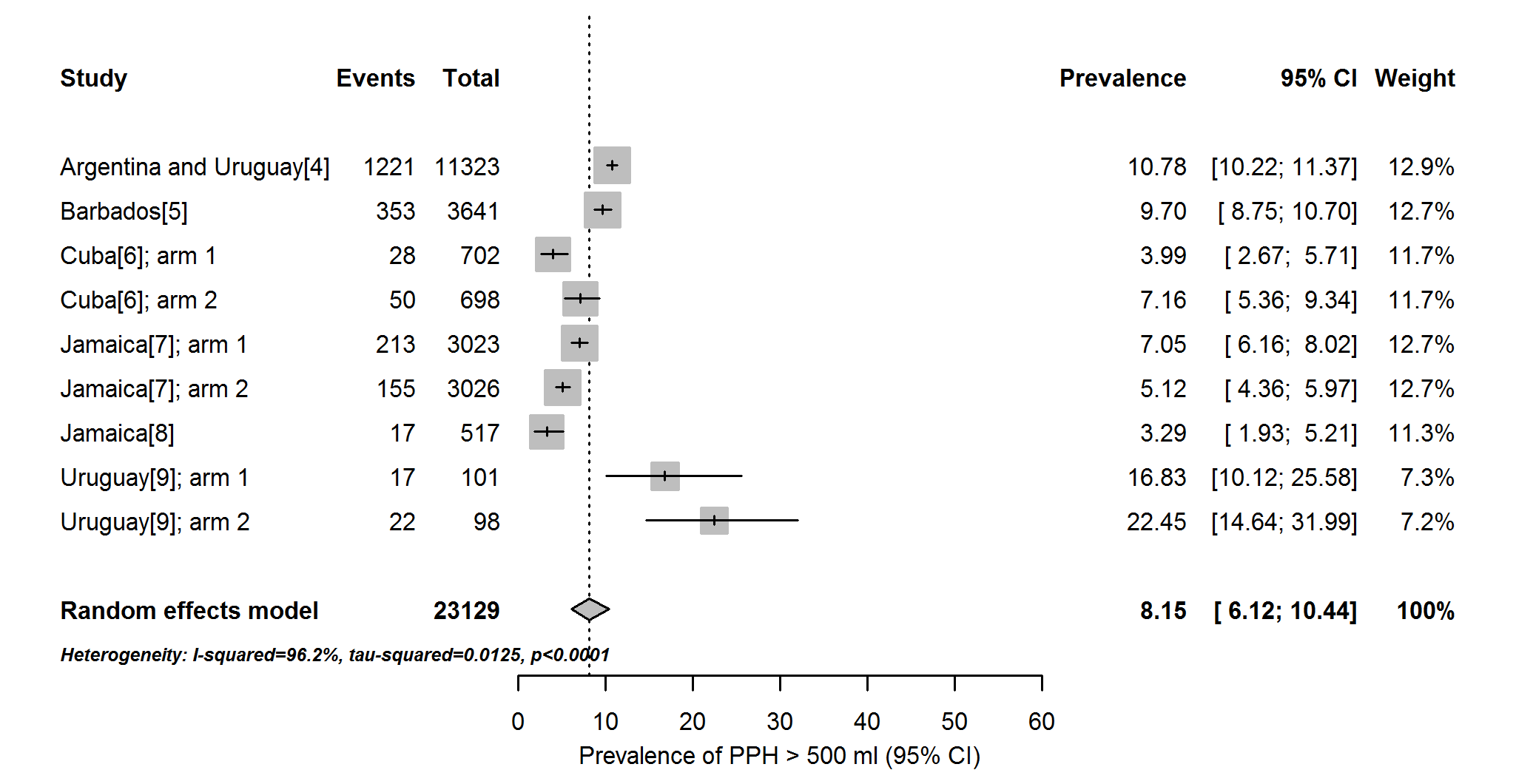

Supplement: Figure S2 — Forest plot of prevalence of PPH≥500 ml amongst studies conducted in Latin America and the Caribbean. (TIFF) [file pone.0041114.s002.tiff]

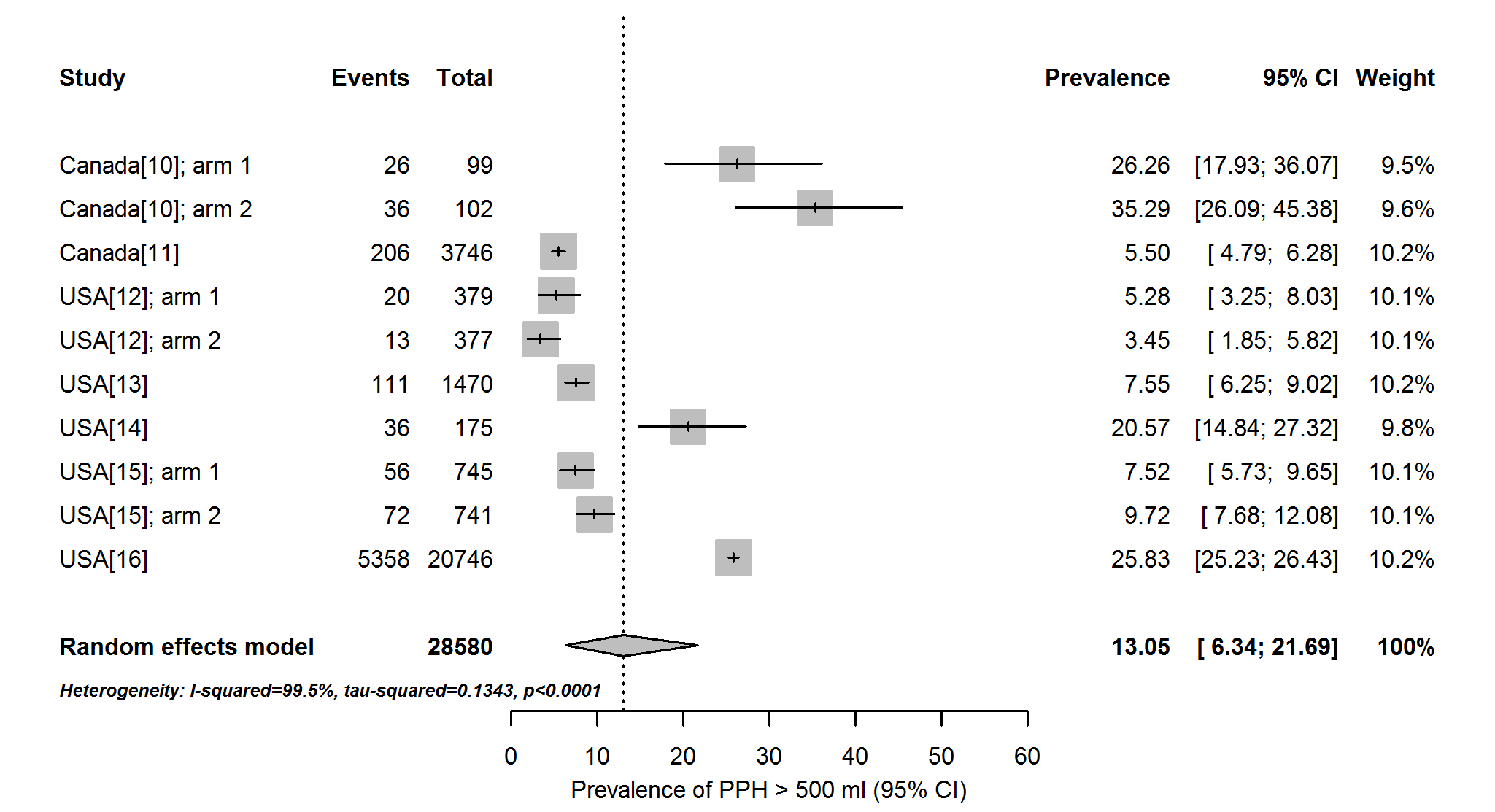

Supplement: Figure S3 — Forest plot of prevalence of PPH≥500 ml amongst studies conducted in Northern America. (TIFF) [file pone.0041114.s003.tiff]

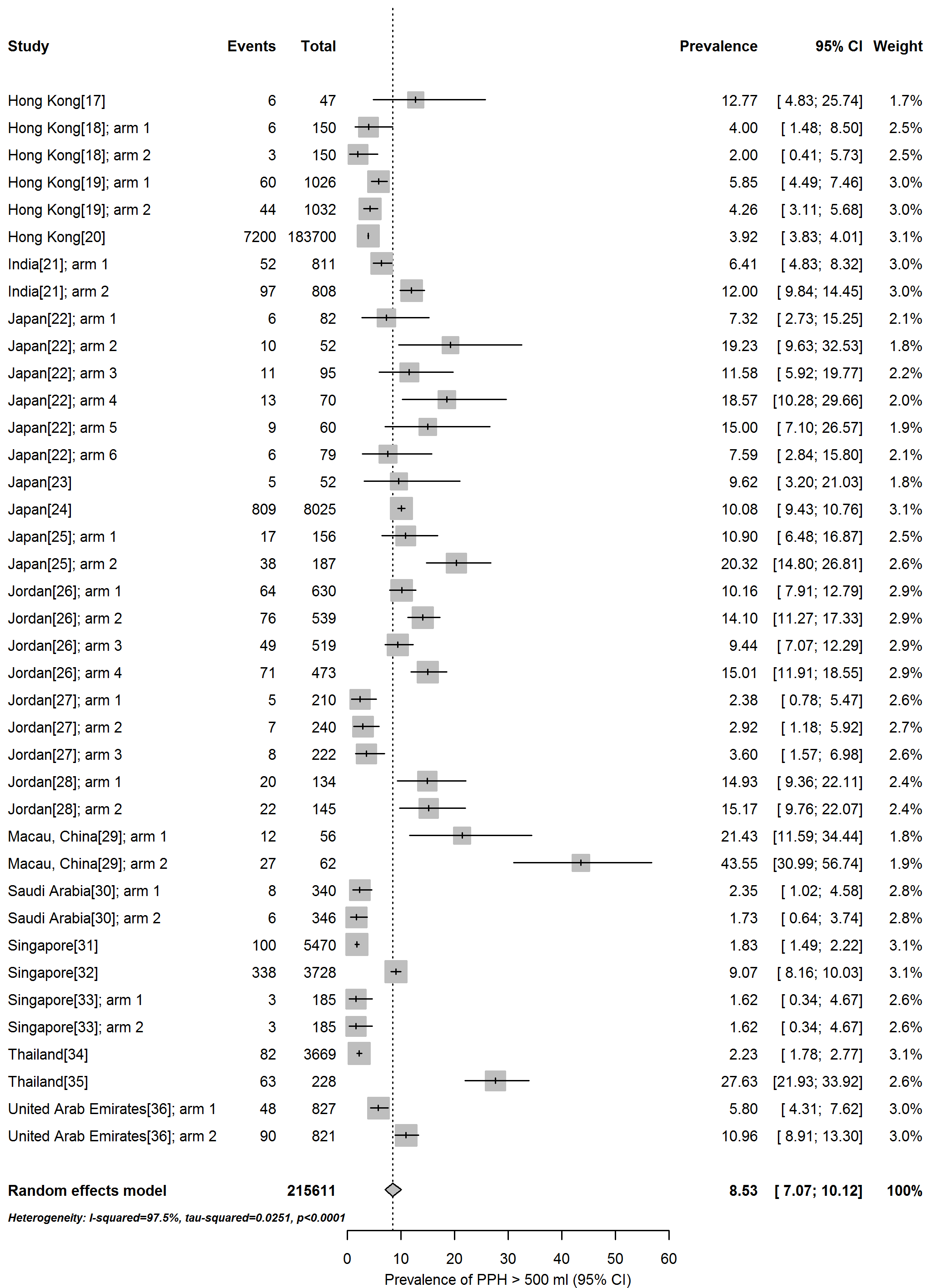

Supplement: Figure S4 — Forest plot of prevalence of PPH≥500 ml amongst studies conducted in Asia. (TIFF) [file pone.0041114.s004.tiff]

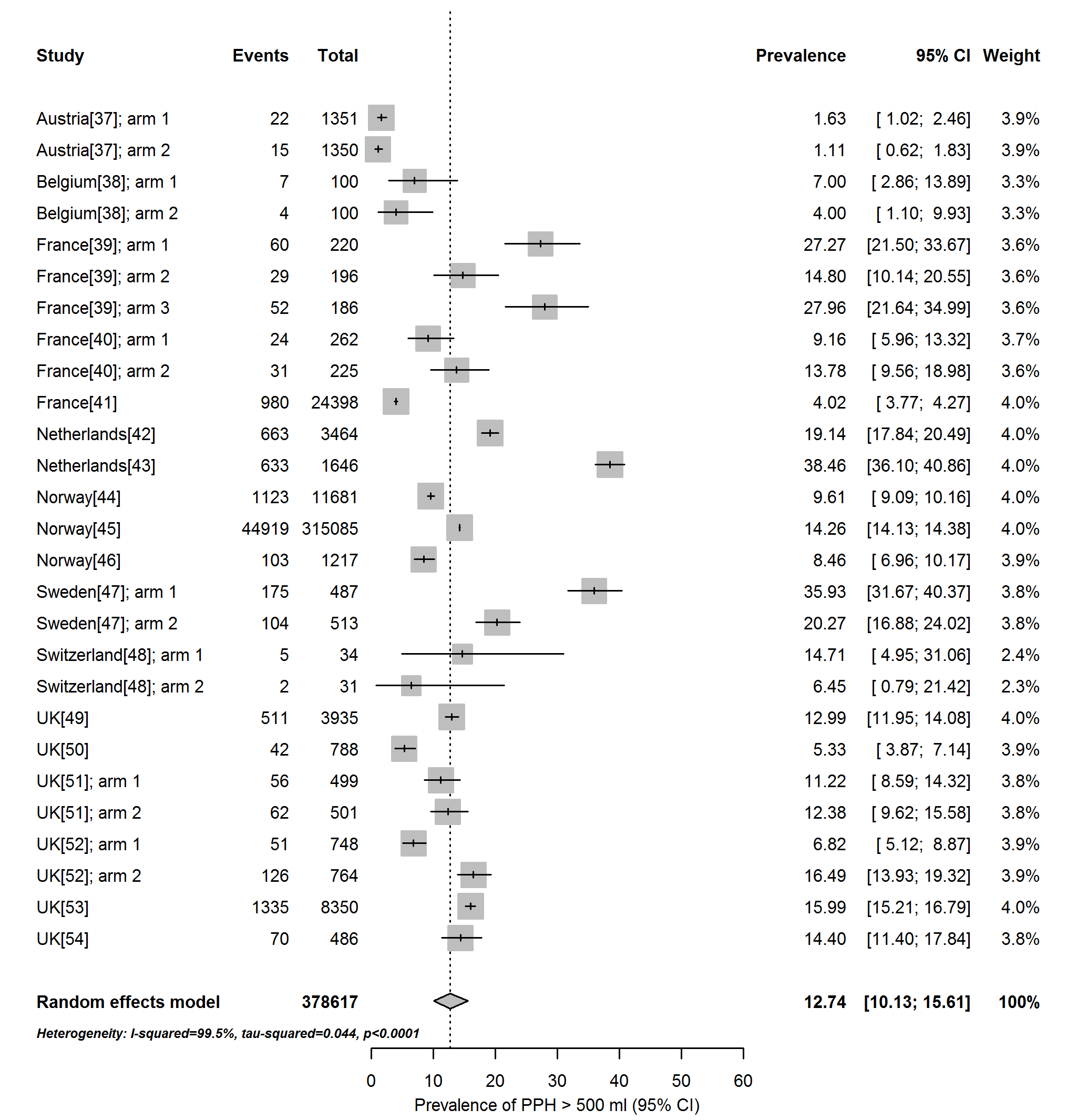

Supplement: Figure S5 — Forest plot of prevalence of PPH≥500 ml amongst studies conducted in Europe. (TIFF) [file pone.0041114.s005.tiff]

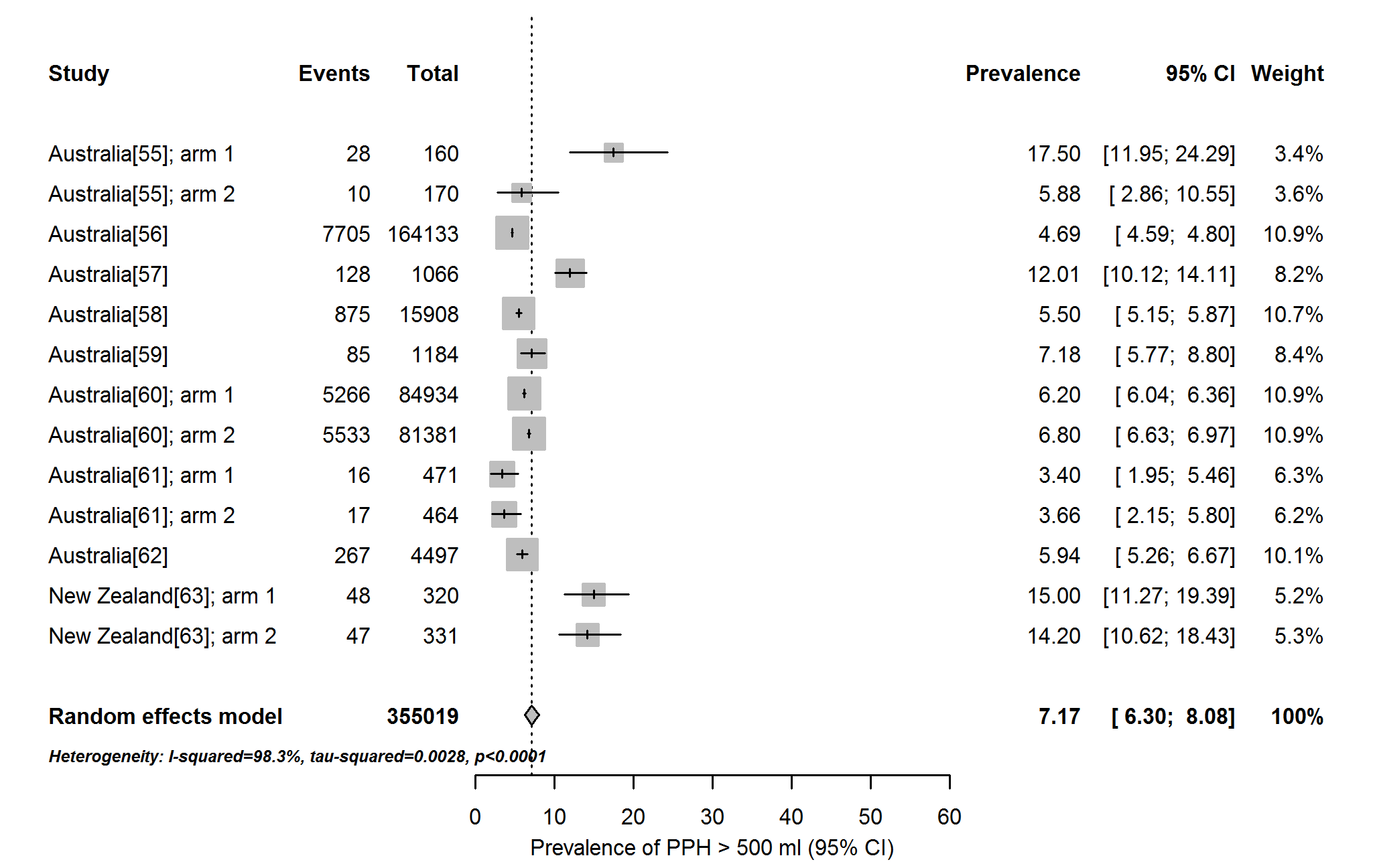

Supplement: Figure S6 — Forest plot of prevalence of PPH≥500 ml amongst studies conducted in Oceania. (TIFF) [file pone.0041114.s006.tiff]

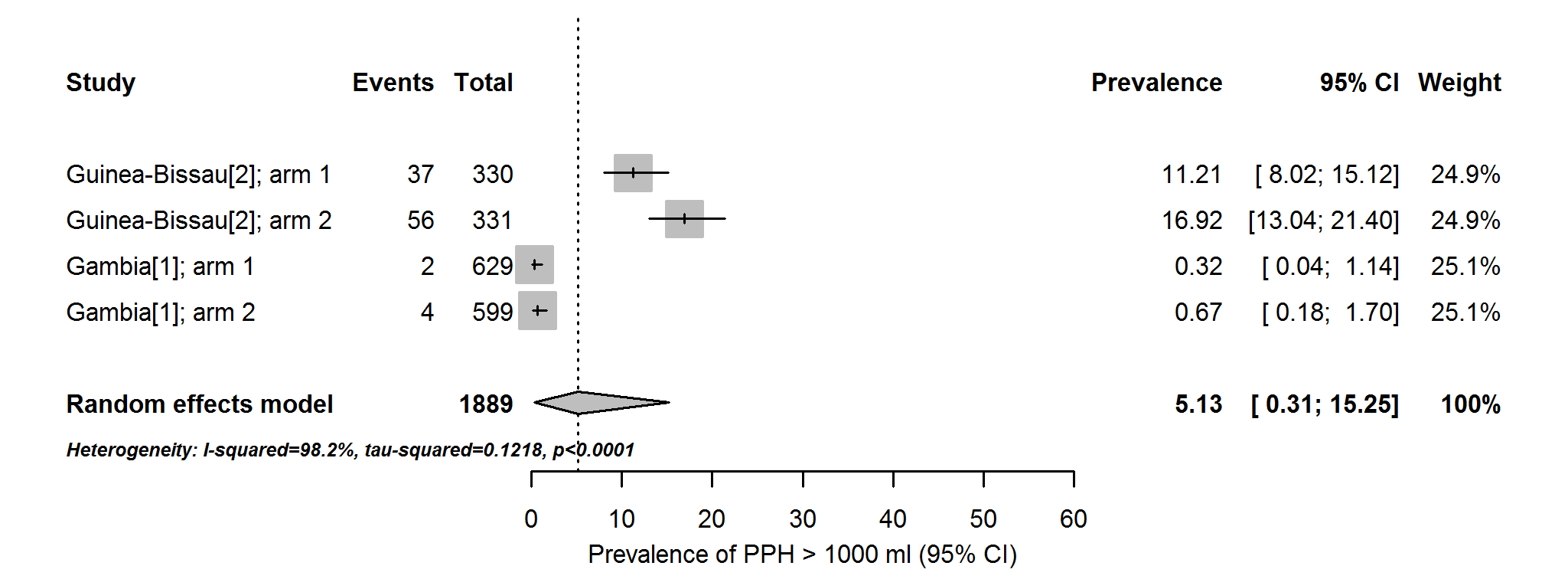

Supplement: Figure S7 — Forest plot of prevalence of PPH≥1000 ml amongst studies conducted in Africa. (TIFF) [file pone.0041114.s007.tiff]

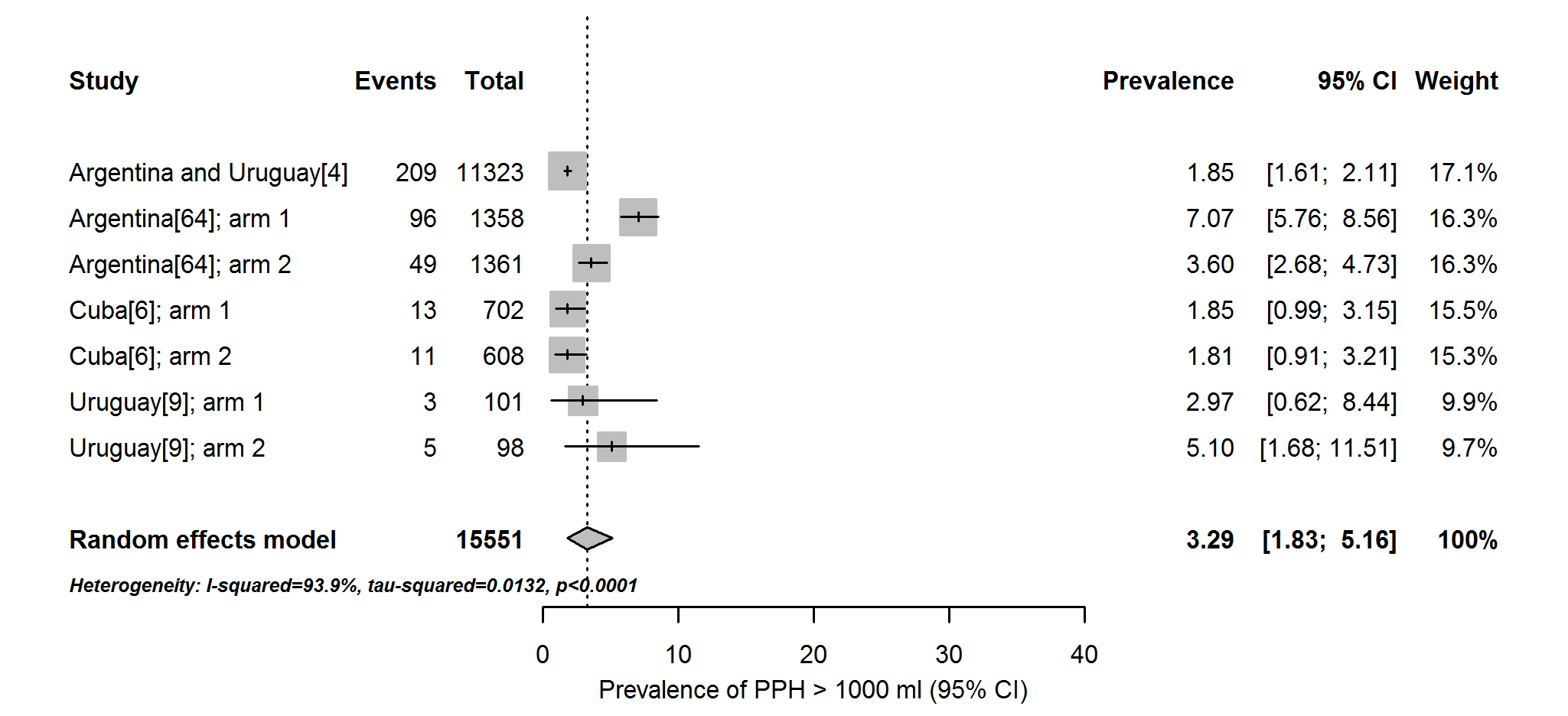

Supplement: Figure S8 — Forest plot of prevalence of PPH≥1000 ml amongst studies conducted in Latin America and the Caribbean. (TIFF) [file pone.0041114.s008.tiff]

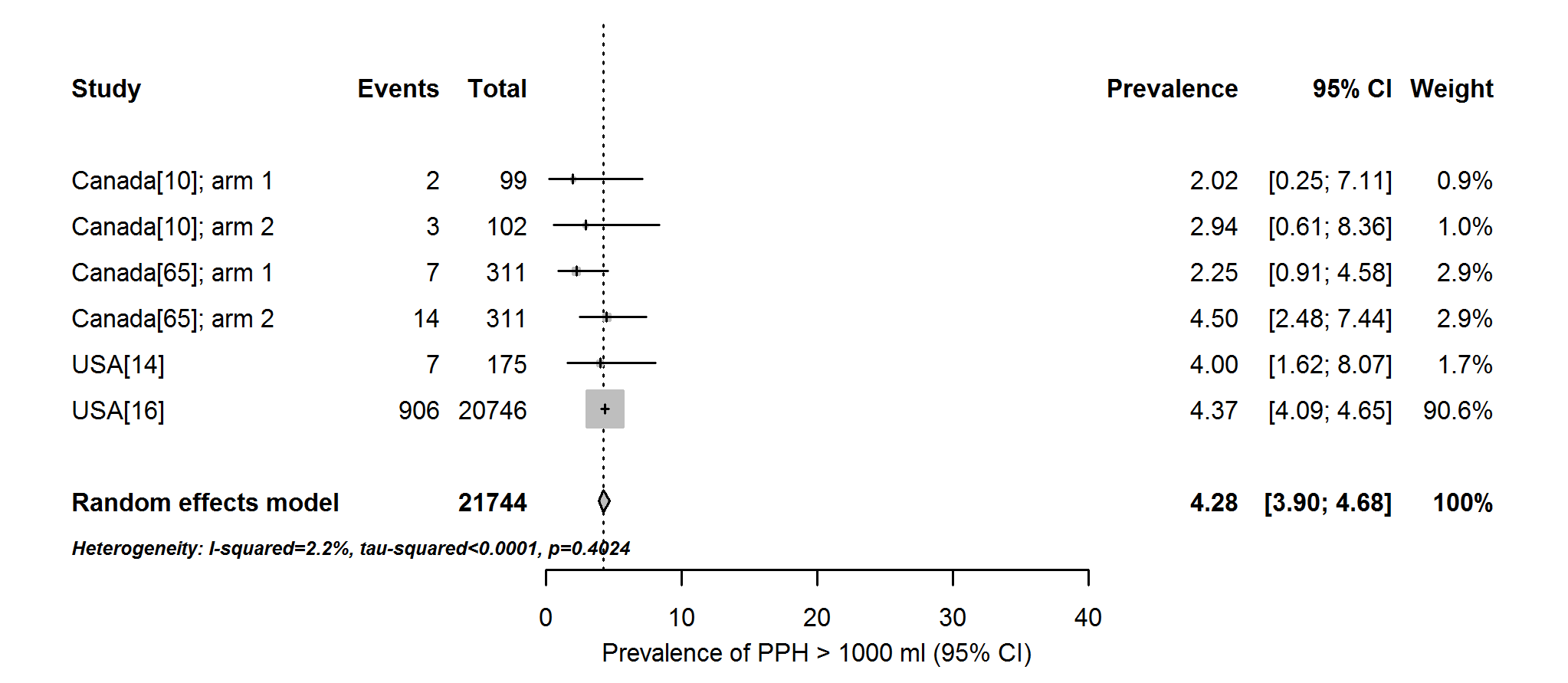

Supplement: Figure S9 — Forest plot of prevalence of PPH≥1000 ml amongst studies conducted in Northern America. (TIFF) [file pone.0041114.s009.tiff]

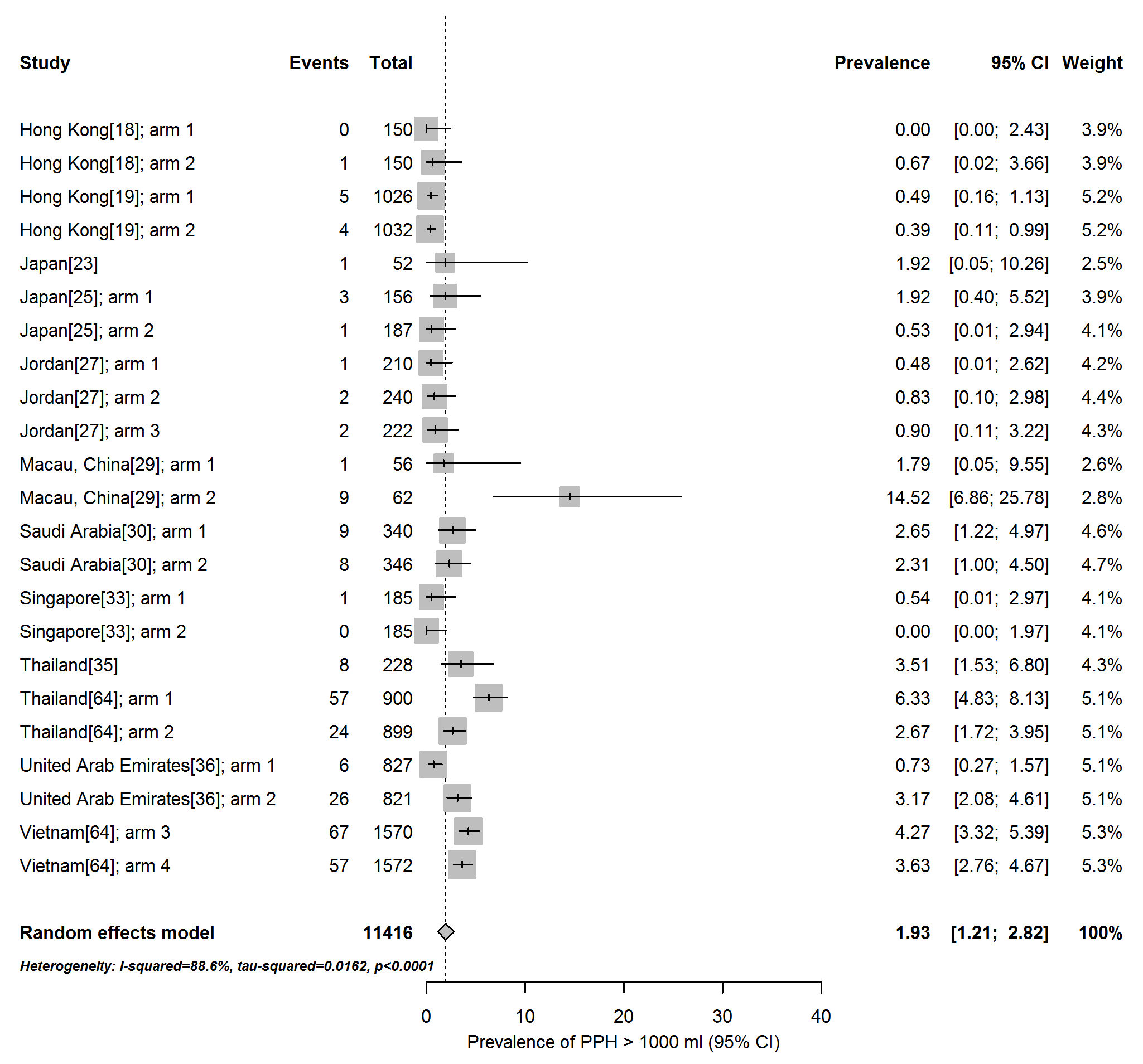

Supplement: Figure S10 — Forest plot of prevalence of PPH≥1000 ml amongst studies conducted in Asia. (TIFF) [file pone.0041114.s010.tiff]

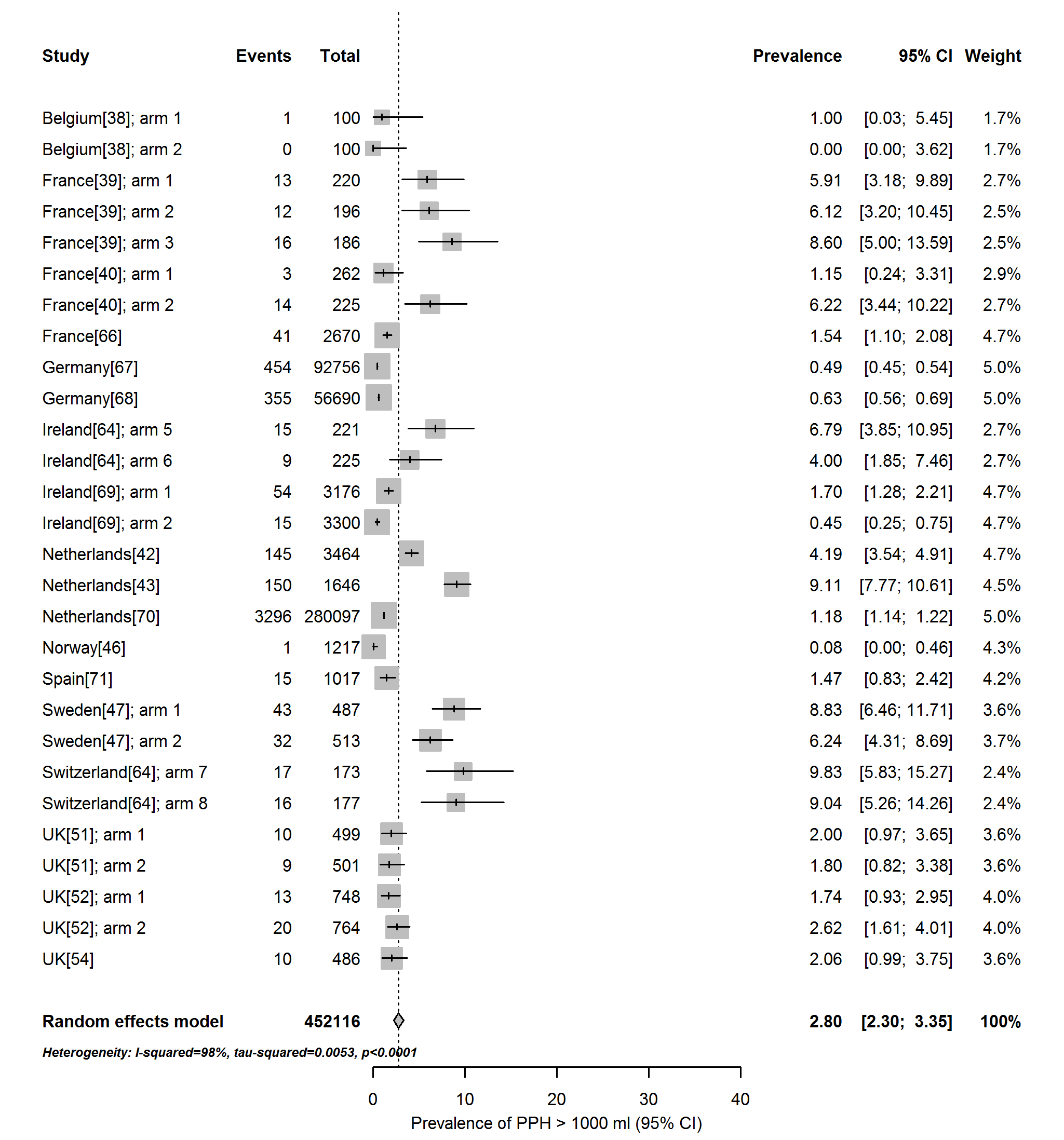

Supplement: Figure S11 — Forest plot of prevalence of PPH≥1000 ml amongst studies conducted in Europe. (TIFF) [file pone.0041114.s011.tiff]

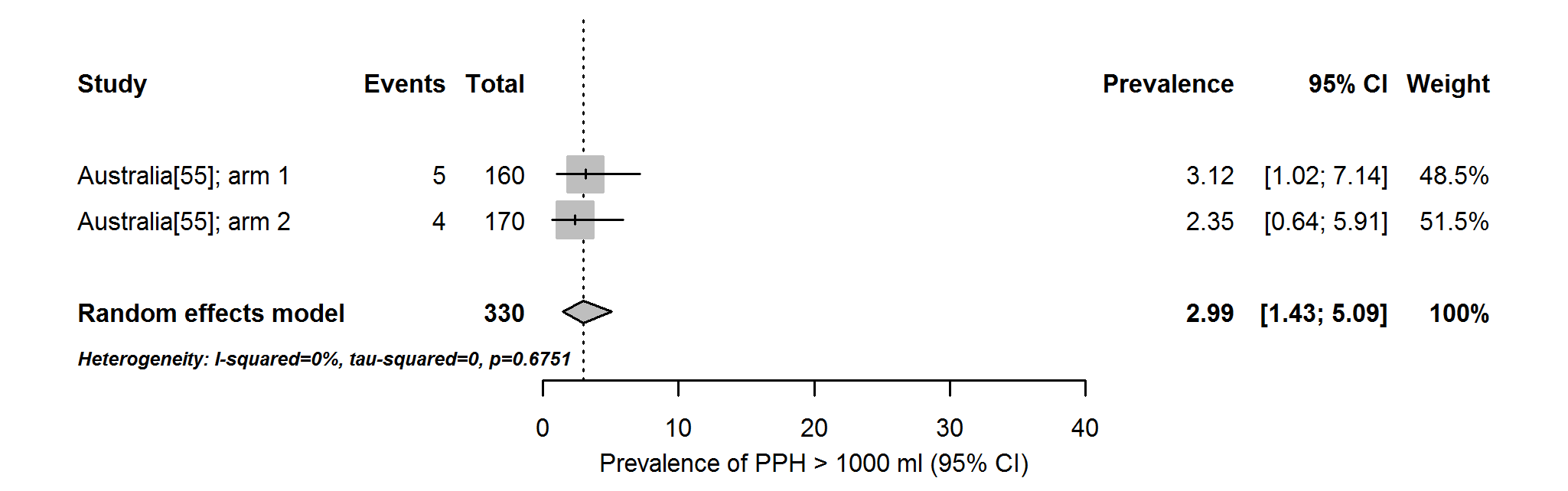

Supplement: Figure S12 — Forest plot of prevalence of PPH≥1000 ml amongst studies conducted in Oceania. (TIFF) [file pone.0041114.s012.tiff]
