# Supplementary material for: Identifying Regional Variation in the Prevalence of Postpartum Haemorrhage: A Systematic Review and Meta-Analysis
Source: PLoS One. 2012 Jul 23;7(7):e41114. doi: 10.1371/journal.pone.0041114 (PMC3402540; doi:10.1371/journal.pone.0041114)
Supplement: Table S1 — Prevalence of PPH≥500 ml by UN regions. (DOCX) [file pone.0041114.s013.docx]

**Table S1. Prevalence of PPH ≥ 500 ml by UN regions**

| **Group/Sub-Group** | **No. articles** | **No. datasets** | **No. women** | **No. women with blood loss ≥ 500 ml** | **% min** | **% max** | **I^2^** | **P-Value^1^** | **Prevalence of blood loss ≥ 500ml per 100** |
| --- | --- | --- | --- | --- | --- | --- | --- | --- | --- |
| **Africa** | 3 | 6 | 2,738 | 645 | 11.0 | 51.4 | 98.4 | <0.001 | 25.7 (13.9-39.7) |
| Eastern Africa | 1 | 2 | 849 | 184 | 18.5 | 24.5 | 77.5 | 0.03 | 21.5 (16.0-27.6) |
| Middle Africa | 0 | 0 | - | - | - | - | - | - | - |
| Southern Africa | 0 | 0 | - | - | - | - | - | - | - |
| Northern Africa | 0 | 0 | - | - | - | - | - | - | - |
| Western Africa | 2 | 4 | 1,889 | 461 | 11.0 | 51.4 | 99 | <0.001 | 28.0 (10.2-50.5) |
| **Latin America and the Caribbean** | 6 | 9 | 23,129 | 2,076 | 3.3 | 22.5 | 96.2 | <0.001 | 8.2 (6.1-10.4) |
| Caribbean | 4 | 6 | 11,607 | 816 | 3.3 | 9.7 | 93.7 | <0.001 | 6.0 (4.3-8.0) |
| Central America | 0 | 0 | - | - | - | - | - | - | - |
| South America | 2 | 3 | 11,522 | 1,260 | 10.8 | 22.5 | 85.4 | 0.001 | 16.0 (9.0-24.5) |
| **Northern America** | 7 | 10 | 28,580 | 5,934 | 3.5 | 35.3 | 99.5 | <0.001 | 13.1 (6.3-21.7) |
| **Asia** | 20 | 39 | 215,611 | 9,494 | 1.6 | 43.6 | 97.5 | <0.001 | 8.5 (7.1-10.1) |
| Central Asia | 0 | 0 | - | - | - | - | - | - | - |
| Eastern Asia | 12 | 22 | 204,649 | 8,726 | 1.6 | 43.6 | 97.8 | <0.001 | 8.8 (6.8-11.0) |
| Southern Asia | 1 | 2 | 1,619 | 149 | 6.4 | 12.0 | 93.5 | <0.001 | 9.1 (4.4-15.3) |
| South-Eastern Asia | 2 | 2 | 3,397 | 145 | 2.2 | 27.6 | 99.3 | <0.001 | 11.8 (0.2-46.2) |
| Western Asia | 5 | 13 | 5,446 | 474 | 1.7 | 15.2 | 92.7 | <0.001 | 7.6 (5.2-10.5) |
| **Europe** | 18 | 27 | 378,617 | 51,204 | 1.1 | 38.5 | 99.5 | <0.001 | 12.7 (10.1-15.6) |
| Eastern Europe | 0 | 0 | - | - | - | - | - | - | - |
| Northern Europe | 10 | 13 | 345,054 | 48,677 | 5.3 | 35.9 | 97.9 | <0.001 | 13.4 (11.5-15.4) |
| Southern Europe | 0 | 0 | - | - | - | - | - | - | - |
| Western Europe | 8 | 14 | 33,563 | 2,527 | 1.1 | 38.5 | 99.4 | <0.001 | 12.0 (6.1-19.5) |
| **Oceania** | 9 | 13 | 355,019 | 20,025 | 3.4 | 17.5 | 98.3 | <0.001 | 7.2 (6.3-8.1) |
| Australia and New Zealand | 9 | 13 | 355,019 | 20,025 | 3.4 | 17.5 | 98.3 | <0.001 | 7.2 (6.3-8.1) |
| Melanesia | 0 | 0 | - | - | - | - | - | - | - |
| Micronesia | 0 | 0 | - | - | - | - | - | - | - |
| Polynesia | 0 | 0 | - | - | - | - | - | - | - |

^1^From test of heterogeneity
