# Supplementary material for: Identifying Regional Variation in the Prevalence of Postpartum Haemorrhage: A Systematic Review and Meta-Analysis
Source: PLoS One. 2012 Jul 23;7(7):e41114. doi: 10.1371/journal.pone.0041114 (PMC3402540; doi:10.1371/journal.pone.0041114)
Supplement: Table S2 — Prevalence of PPH≥1000 ml by UN regions. (DOCX) [file pone.0041114.s014.docx]

**Table S2. Prevalence of PPH ≥ 1000 ml by UN regions**

| **Group/Sub-Group** | **No. articles** | **No. datasets** | **No. women** | **No. women with blood loss ≥ 1000 ml** | **% min** | **% max** | **I^2^** | **P-Value^1^** | **Prevalence of blood loss ≥ 1000 ml per 100** |
| --- | --- | --- | --- | --- | --- | --- | --- | --- | --- |
| **Africa** | 2 | 4 | 1,889 | 99 | 0.3 | 16.9 | 98.2 | <0.001 | 5.1 (0.3-15.3) |
| Eastern Africa | 0 | 0 | - | - | - | - | - | - | - |
| Middle Africa | 0 | 0 | - | - | - | - | - | - | - |
| Southern Africa | 0 | 0 | - | - | - | - | - | - | - |
| Northern Africa | 0 | 0 | - | - | - | - | - | - | - |
| Western Africa | 2 | 4 | 1,889 | 99 | 0.3 | 16.9 | 98.2 | <0.001 | 5.1 (0.3-15.3) |
| **Latin America and the Caribbean** | 4 | 7 | 15,551 | 386 | 1.6 | 7.1 | 93.9 | <0.001 | 3.3 (1.8-5.2) |
| Caribbean | 1 | 2 | 1,310 | 24 | 1.6 | 1.9 | 0 | 0.97 | 1.9 (1.2-2.7) |
| Central America | 0 | 0 | - | - | - | - | - | - | - |
| South America | 3 | 5 | 14,241 | 362 | 1.9 | 7.1 | 95.9 | <0.001 | 4.1 (3.9-4.68) |
| **Northern America** | 4 | 6 | 21,744 | 939 | 1.6 | 5.3 | 2.2 | 0.40 | 4.3 (4.1-4.6) |
| **Asia** | 12 | 23 | 11,416 | 293 | 0 | 14.5 | 88.6 | <0.001 | 1.9 (1.2-2.8) |
| Central Asia | 0 | 0 | - | - | - | - | - | - | - |
| Eastern Asia | 6 | 11 | 3,241 | 26 | 0 | 14.5 | 71.6 | <0.001 | 1.1 (0.5 -2.1) |
| Southern Asia | 0 | 0 | - | - | - | - | - | - | - |
| South-Eastern Asia | 2 | 5 | 5,169 | 213 | 2.7 | 6.3 | 74.8 | 0.003 | 4.1 (3.0-5.3) |
| Western Asia | 3 | 7 | 3,006 | 54 | 0.5 | 3.2 | 70.1 | 0.003 | 1.7 (0.9-2.6) |
| **Europe** | 18 | 28 | 452,116 | 4,779 | 0 | 9.8 | 98.0 | <0.001 | 2.8 (2.3-3.4) |
| Eastern Europe | 0 | 0 | - | - | - | - | - | - | - |
| Northern Europe | 7 | 12 | 12,137 | 231 | 0.1 | 8.8 | 94.8 | <0.001 | 2.7 (1.5-4.1) |
| Southern Europe | 1 | 1 | 1,017 | 15 | 1.5 | 1.5 | n/a | n/a | 1.5 (0.9-2.4) |
| Western Europe | 10 | 15 | 438,962 | 4,533 | 0 | 9.8 | 98.7 | <0.001 | 3.2 (2.5-4.0) |
| **Oceania** | 1 | 2 | 330 | 9 | 2.4 | 3.1 | 0 | 0.68 | 3.0 (1.4-5.1) |
| Australia and New Zealand | 1 | 2 | 330 | 9 | 2.4 | 3.1 | 0 | 0.68 | 3.0 (1.4-5.1) |
| Melanesia | 0 | 0 | - | - | - | - | - | - | - |
| Micronesia | 0 | 0 | - | - | - | - | - | - | - |
| Polynesia | 0 | 0 | - | - | - | - | - | - | - |

^1^From test of heterogeneity
