# Supplementary material for: Identifying Regional Variation in the Prevalence of Postpartum Haemorrhage: A Systematic Review and Meta-Analysis
Source: PLoS One. 2012 Jul 23;7(7):e41114. doi: 10.1371/journal.pone.0041114 (PMC3402540; doi:10.1371/journal.pone.0041114)
Supplement: File S1 — (DOCX) [file pone.0041114.s015.docx]

Studies included in the review

1. Walraven G, Blum J, Dampha Y, Sowe M, Morison L, Winikoff B, et al. Misoprostol in the management of the third stage of labour in the home delivery setting in rural Gambia: a randomised controlled trial. BJOG: An International Journal of Obstetrics & Gynaecology. 2005;112(9):1277-83.

2. Hoj L, Cardoso P, Nielsen BB, Hvidman L, Nielsen J, Aaby P. Effect of sublingual misoprostol on severe postpartum haemorrhage in a primary health centre in Guinea-Bissau: Randomised double blind clinical trial. British Medical Journal. 2005;331(7519):723-7.

3. Prata N, Mbaruku G, Campbell M, Potts M, Vahidnia F. Controlling postpartum hemorrhage after home births in Tanzania. International Journal of Gynecology & Obstetrics. 2005;90(1):51-5.

4. Sosa CG, Althabe F, Belizan JM, Buekens P. Risk factors for postpartum hemorrhage in vaginal deliveries in a Latin-American population. Obstetrics & Gynecology. 2009;113(6):1313-9.

5. Rotchell YE, Cruickshank JK, Gay MP, Griffiths J, Stewart A, Farrell B, et al. Barbados Low Dose Aspirin Study in Pregnancy (BLASP): a randomised trial for the prevention of pre-eclampsia and its complications.[see comment]. British Journal of Obstetrics & Gynaecology. 1998;105(3):286-92.

6. Carbonell i Esteve JL, Hernandez JMR, Piloto M, Setien SA, Texido CS, Tomasi G, et al. Active management of the third phase of labour plus 400 mug of sublingual misoprostol and 200 mug of rectal misoprostol versus active management only in the prevention of post-partum haemorrhage. A randomised clinical trial. [Spanish]. Progresos de Obstetricia y Ginecologia. 2009;52(10):543-51.

7. Golding J. A randomised trial of low dose aspirin for primiparae in pregnancy. The Jamaica Low Dose Aspirin Study Group.[see comment]. British Journal of Obstetrics & Gynaecology. 1998;105(3):293-9.

8. Phillip H, Fletcher H, Reid M. The impact of induced labour on postpartum blood loss. Journal of Obstetrics & Gynaecology. 2004;24(1):12-5.

9. Althabe F, Aleman A, Tomasso G, Gibbons L, Vitureira G, Belizan JM, et al. A pilot randomized controlled trial of controlled cord traction to reduce postpartum blood loss. International Journal of Gynecology and Obstetrics. 2009;107(1):4-7.

10. Davies GAL, Tessier JL, Woodman MC, Lipson A, Hahn PM. Maternal hemodynamics after oxytocin bolus compared with infusion in the third stage of labor: A randomized controlled trial. Obstetrics and Gynecology. 2005;105(2):294-9.

11. Delaney T, Young DC. Spontaneous Versus Induced Labor After a Previous Cesarean Delivery. Obstetrics & Gynecology. 2003;102(1):39-44.

12. Bhullar A, Carlan SJ, Hamm J, Lamberty N, White L, Richichi K. Buccal misoprostol to decrease blood loss after vaginal delivery: a randomized trial. Obstetrics & Gynecology. 2004;104(6):1282-8.

13. Dugan-Kim M, Connell S, Stika C, Wong CA, Gossett DR. Epistaxis of pregnancy and association with postpartum hemorrhage. Obstetrics & Gynecology. 2009;114(6):1322-5.

14. Fenton JJ, Baumeister LM, Fogarty J. Active management of the third stage of labor among American Indian women. Family Medicine. 2005;37(6):410-4.

15. Jackson Jr KW, Allbert JR, Schemmer GK, Elliot M, Humphrey A, Taylor J. A randomized controlled trial comparing oxytocin administration before and after placental delivery in the prevention of postpartum hemorrhage. American Journal of Obstetrics and Gynecology. 2001;185(4):873-7.

16. Lu MC, Korst LM, Fridman M, Muthengi E, Gregory KD. Identifying women most likely to benefit from prevention strategies for postpartum hemorrhage. Journal of Perinatology. 2009;29(6):422-7.

17. Chan SM, Nelson EAS, Leung SSF, Li CY. Postnatal iron status of Hong Kong Chinese women in a longitudinal study of maternal nutrition. European Journal of Clinical Nutrition. 2001;55(7):538-46.

18. Leung SW, Ng PS, Wong WY, Cheung TH. A randomised trial of carbetocin versus syntometrine in the management of the third stage of labour. BJOG: An International Journal of Obstetrics & Gynaecology. 2006;113(12):1459-64.

19. Ng PS, Chan AS, Sin WK, Tang LC, Cheung KB, Yuen PM. A multicentre randomized controlled trial of oral misoprostol and i.m. syntometrine in the management of the third stage of labour. Human Reproduction. 2001;16(1):31-5.

20. Yong SPY, Cheung KB. Management of primary postpartum haemorrhage with arterial embolisation in Hong Kong public hospitals. Hong Kong Medical Journal. 2006;12(6):437-41.

21. Geller SE, Goudar SS, Adams MG, Naik VA, Patel A, Bellad MB, et al. Factors associated with acute postpartum hemorrhage in low-risk women delivering in rural India.[see comment]. International Journal of Gynaecology & Obstetrics. 2008;101(1):94-9.

22. Fujimoto M, Takeuchi K, Sugimoto M, Maruo T. Prevention of postpartum hemorrhage by uterotonic agents: comparison of oxytocin and methylergometrine in the management of the third stage of labor. Acta Obstetricia et Gynecologica Scandinavica. 2006;85(11):1310-4.

23. Matsubara S, Ohkuchi A, Kikkawa M, Izumi A, Kuwata T, Usui R, et al. Blood loss in low-lying placenta: placental edge to cervical internal os distance of less vs. more than 2 cm. Journal of Perinatal Medicine. 2008;36(6):507-12.

24. Ohkuchi A, Onagawa T, Usui R, Koike T, Hiratsuka M, Izumi A, et al. Effect of maternal age on blood loss during parturition: a retrospective multivariate analysis of 10,053 cases. Journal of Perinatal Medicine. 2003;31(3):209-15.

25. Saito K, Haruki A, Ishikawa H, Takahashi T, Nagase H, Koyama M, et al. Prospective study of intramuscular ergometrine compared with intramuscular oxytocin for prevention of postpartum hemorrhage. Journal of Obstetrics & Gynaecology Research. 2007;33(3):254-8.

26. Abou Omar AA. Prevention of postpartum hemorrhage, safety and efficacy. Saudi Med J 2001;22 (12):1118-21.

27. Al Momani M. Is treatment with oral or rectal misoprostol effective to prevent postpartum hemorrhage? J Arab Board Med Special 2006;8(2):110-4.

28. Malkawi H, Hindawi I, Hiasat M, Amarin V. Oxytocin and syntometrine in prevention of postpartum hemorrhage. 2005.

29. Ngan L, Keong W, Martins R. Carbetocin versus a combination of oxytocin and ergometrine in control of postpartum blood loss. International Journal of Gynaecology & Obstetrics. 2007;97(2):152-3.

30. Rashid M, Clark A, Rashid MH. A randomised controlled trial comparing the efficacy of intramuscular syntometrine and intravenous syntocinon, in preventing postpartum haemorrhage. Journal of Obstetrics & Gynaecology. 2009;29(5):396-401.

31. Koh E, Devendra K, Tan LK. B-Lynch suture for the treatment of uterine atony. Singapore Medical Journal. 2009;50(7):693-7.

32. Singh K, Fong YF, Arulkumaran S. Anaemia in pregnancy--a cross-sectional study in Singapore. European Journal of Clinical Nutrition. 1998;52(1):65-70. Epub 1998/03/03.

33. Su LL, Rauff M, Chan YH, Mohamad Suphan N, Lau TP, Biswas A, et al. Carbetocin versus syntometrine for the third stage of labour following vaginal delivery - A double-blind randomised controlled trial. BJOG: An International Journal of Obstetrics and Gynaecology. 2009;116(11):1461-6.

34. Liabsuetrakul T, Promvijit T, Pattanapisalsak C, Silalai S, Ampawa T. A criterion-based obstetric morbidity audit in southern Thailand. International Journal of Gynaecology & Obstetrics. 2008;103(2):166-71.

35. Prasertcharoensuk W, Swadpanich U, Lumbiganon P. Accuracy of the blood loss estimation in the third stage of labor. International Journal of Gynaecology & Obstetrics. 2000;71(1):69-70.

36. Khan GQ, John IS, Wani S, Doherty T, Sibai BM. Controlled cord traction versus minimal intervention techniques in delivery of the placenta: a randomized controlled trial. American Journal of Obstetrics & Gynecology. 1997;177(4):770-4.

37. Bodner-Adler B, Bodner K, Kimberger O, Lozanov P, Husslein P, Mayerhofer K. Influence of the birth attendant on maternal and neonatal outcomes during normal vaginal delivery: a comparison between midwife and physician management.[see comment]. Wiener Klinische Wochenschrift. 2004;116(11-12):379-84.

38. Amant F, Spitz B, Timmerman D, Corremans A, Van Assche FA. Misoprostol compared with methylergometrine for the prevention of postpartum haemorrhage: a double–blind randomised trial. BJOG: An International Journal of Obstetrics & Gynaecology. 1999;106(10):1066-70.

39. Benchimol M, Gondry J, Mention J, al. e. Role of misoprostol in controlled delivery [Place du misoprostol dans la direction de la delivrance]. J Gynecol Obstet Biol Reprod (Paris). 2001;30(6):576-83.

40. Brement S, Mossan S, Belery A, Racinet C. [Delivery in lateral position. Randomized clinical trial comparing the maternal positions in lateral position and dorsal position for the second stage of labour]. Gynecologie, Obstetrique & Fertilite. 2007;35(7-8):637-44.

41. Chauleur C, Fanget C, Tourne G, Levy R, Larchez C, Seffert P. Serious primary post-partum hemorrhage, arterial embolization and future fertility: a retrospective study of 46 cases. Human Reproduction. 2008;23(7):1553-9.

42. Bais JMJ, Eskes M, Pel M, Bonsel GJ, Bleker OP. Postpartum haemorrhage in nulliparous women: incidence and risk factors in low and high risk women. A Dutch population-based cohort study on standard (> or = 500 ml) and severe (> or = 1000 ml) postpartum haemorrhage. European Journal of Obstetrics, Gynecology, & Reproductive Biology. 2004;115(2):166-72.

43. de Jonge A, van Diem MT, Scheepers PLH, van der Pal-de Bruin KM, Lagro-Janssen ALM. Increased blood loss in upright birthing positions originates from perineal damage. BJOG: An International Journal of Obstetrics & Gynaecology. 2007;114(3):349-55.

44. Eggebo TM, Gjessing LK. [Hemorrhage after vaginal delivery].[see comment]. Tidsskrift for Den Norske Laegeforening. 2000;120(24):2860-3. Blodning etter vaginal fodsel.

45. Eskild A, Vatten LJ. Abnormal bleeding associated with preeclampsia: a population study of 315,085 pregnancies. Acta Obstetricia et Gynecologica Scandinavica. 2009;88(2):154-8.

46. Schmidt N, Abelsen B, Oian P. Deliveries in maternity homes in Norway: Results from a 2-year prospective study. Acta Obstetricia et Gynecologica Scandinavica. 2002;81(8):731-7.

47. Nordström L, Fogelstam K, Fridman G, Larsson A, Rydhstroem H. Routine oxytocin in the third stage of labour: a placebo controlled randomised trial. BJOG: An International Journal of Obstetrics & Gynaecology. 1997;104(7):781-6.

48. Surbek DV, Fehr PM, Hosli I, Holzgreve W. Oral Misoprostol for Third Stage of Labor: A Randomized Placebo-Controlled Trial. Obstetrics & Gynecology. 1999;94(2):255-8.

49. Alfirevic Z, Edwards G, Platt MJ. The impact of delivery suite guidelines on intrapartum care in 'standard primigravida'. European Journal of Obstetrics, Gynecology, & Reproductive Biology. 2004;115(1):28-31.

50. Bugg GJ, Atwal GS, Maresh M. Grandmultiparae in a modern setting. BJOG: An International Journal of Obstetrics & Gynaecology. 2002;109(3):249-53.

51. El-Refaey H, Nooh R, O'Brien P, Abdalla M, Geary M, Walder J, et al. The misoprostol third stage of labour study: a randomised controlled comparison between orally administered misoprostol and standard management. BJOG: An International Journal of Obstetrics & Gynaecology. 2000;107(9):1104-10.

52. Rogers J, Wood J, McCandlish R, Ayers S, Truesdale A, Elbourne D. Active versus expectant management of third stage of labour: the Hinchingbrooke randomised controlled trial. The Lancet. 1998;351(9104):693-9.

53. Usha Kiran TS, Hemmadi S, Bethel J, Evans J. Outcome of pregnancy in a woman with an increased body mass index. BJOG: An International Journal of Obstetrics & Gynaecology. 2005;112(6):768-72.

54. Marchant S, Alexander J, Thomas P, Garcia J, Brocklehurst P, Keene J. Risk factors for hospital admission related to excessive and/or prolonged postpartum vaginal blood loss after the first 24 h following childbirth. Paediatric and Perinatal Epidemiology. 2006;20(5):392-402.

55. Cook C, Spurrett B, Murray H. A Randomized Clinical Trial Comparing Oral Misoprostol With Synthetic Oxytocin or Syntometrine in the Third Stage of Labour. Australian and New Zealand Journal of Obstetrics and Gynaecology. 1999;39(4):414-9.

56. Ford JB, Roberts CL, Simpson JM, Vaughan J, Cameron CA. Increased postpartum hemorrhage rates in Australia. International Journal of Gynaecology & Obstetrics. 2007;98(3):237-43.

57. Henry A, Birch M-R, Sullivan EA, Katz S, Wang YA. Primary postpartum haemorrhage in an Australian tertiary hospital: a case-control study. Australian & New Zealand Journal of Obstetrics & Gynaecology. 2005;45(3):233-6.

58. Humphrey MD. Is grand multiparity an independent predictor of pregnancy risk? A retrospective observational study. Med J Aust. 2003;179(6):294-6.

59. Lain SJ, Roberts CL, Hadfield RM, Bell JC, Morris JM. How accurate is the reporting of obstetric haemorrhage in hospital discharge data? A validation study. Australian & New Zealand Journal of Obstetrics & Gynaecology. 2008;48(5):481-4.

60. Roberts CL, Ford JB, Algert CS, Bell JC, Simpson JM, Morris JM. Trends in adverse maternal outcomes during childbirth: A population-based study of severe maternal morbidity. BMC Pregnancy and Childbirth. 2009;9(7).

61. Waldenström U, McLachlan H, Forster D, Brennecke S, Brown S. Team midwife care: maternal and infant outcomes. Australian and New Zealand Journal of Obstetrics and Gynaecology. 2001;41(3):257-64.

62. Westerway SC, Keogh J, Heard R, Morris J. Incidence of fetal macrosomia and birth complications in Chinese immigrant women. Australian and New Zealand Journal of Obstetrics and Gynaecology. 2003;43(1):46-9.

63. Sadler LC, Davison T, McCowan LM. A randomised controlled trial and meta-analysis of active management of labour. BJOG: An International Journal of Obstetrics & Gynaecology. 2000;107(7):909-15.

64. Gulmezoglu AM, Villar J, Ngoc NT, Piaggio G, Carroli G, Adetoro L, et al. WHO multicentre randomised trial of misoprostol in the management of the third stage of labour.[see comment]. Lancet. 2001;358(9283):689-95.

65. Baskett TF, Persad VL, Clough HJ, Young DC. Misoprostol versus oxytocin for the reduction of postpartum blood loss. International Journal of Gynaecology & Obstetrics. 2007;97(1):2-5.

66. Shojai R, Desbriere R, Dhifallah S, Courbiere B, Ortega D, D'Ercole C, et al. Rectal misoprostol for postpartum hemorrhage. [French]. Gynecologie Obstetrique Fertilite. 2004;32(9):703-7.

67. David M, Pachaly J, Vetter K, Kentenich H. Birthplace free-standing birth center - Perinatal data in comparison with clinic deliveries in bavaria and berlin. [German]. Zeitschrift fur Geburtshilfe und Neonatologie. 2004;208(3):110-7.

68. Onwuhafua PI. Childbirth in Germany and Nigeria compared. Nigerian Journal of Medicine: Journal of the National Association of Resident Doctors of Nigeria. 2006;15(4):387-92.

69. Rizvi F, Mackey R, Barrett T, McKenna P, Geary M. Successful reduction of massive postpartum haemorrhage by use of guidelines and staff education.[erratum appears in BJOG. 2007 May;114(5):660]. BJOG: An International Journal of Obstetrics & Gynaecology. 2004;111(5):495-8.

70. Amelink-Verburg MP, Verloove-Vanhorick SP, Hakkenberg RMA, Veldhuijzen IME, Bennebroek Gravenhorst J, Buitendijk SE. Evaluation of 280,000 cases in Dutch midwifery practices: a descriptive study. BJOG: An International Journal of Obstetrics & Gynaecology. 2008;115(5):570-8.

71. Corona Gutierrez AA, Teresa Higueras Sanz M, Cabero i Roura L. Short-term complications in patients with cesarean sections. [Spanish]. Progresos en Obstetricia y Ginecologia. 2008;51(12):703-8.
